# Modulating the Fibrillization of Parathyroid-Hormone (PTH) Peptides: Azo-Switches as Reversible and Catalytic Entities

**DOI:** 10.3390/biomedicines10071512

**Published:** 2022-06-26

**Authors:** André Paschold, Bruno Voigt, Gerd Hause, Tim Kohlmann, Sven Rothemund, Wolfgang H. Binder

**Affiliations:** 1 Department of Chemistry, Faculty of Natural Sciences II, Martin-Luther University Halle-Wittenberg, 06120 Halle (Saale), Germany; andre.paschold@chemie.uni-halle.de (A.P.); tim_kohlmann@web.de (T.K.); 2 Department of Physics, Faculty of Natural Sciences II, Martin-Luther University Halle-Wittenberg, 06120 Halle (Saale), Germany; bruno.voigt@physik.uni-halle.de; 3 Biozentrum, Martin-Luther University Halle-Wittenberg, 06120 Halle (Saale), Germany; gerd.hause@biozentrum.uni-halle.de; 4 Core Unit Peptide—Technologies, University Leipzig, 04103 Leipzig, Germany; sven.rothemund@medizin.uni-leipzig.de

**Keywords:** azobenzene, photoswitchable peptides, fibrillization, parathyroid hormone, aggregation

## Abstract

We here report a novel strategy to control the bioavailability of the fibrillizing parathyroid hormone (PTH)-derived peptides, where the concentration of the bioactive form is controlled by an reversible, photoswitchable peptide. PTH_1–84_, a human hormone secreted by the parathyroid glands, is important for the maintenance of extracellular fluid calcium and phosphorus homeostasis. Controlling fibrillization of PTH_1–84_ represents an important approach for in vivo applications, in view of the pharmaceutical applications for this protein. We embed the azobenzene derivate 3-{[(4-aminomethyl)phenyl]diazenyl}benzoic acid (3,4′-AMPB) into the PTH-derived peptide PTH_25–37_ to generate the artificial peptide AzoPTH_25–37_ via solid-phase synthesis. AzoPTH_25–37_ shows excellent photostability (more than 20 h in the dark) and can be reversibly photoswitched between its *cis*/*trans* forms. As investigated by ThT-monitored fibrillization assays, the *trans*-form of AzoPTH_25–37_ fibrillizes similar to PTH_25–37_, while the *cis*-form of AzoPTH_25–37_ generates only amorphous aggregates. Additionally, *cis*-AzoPTH_25–37_ catalytically inhibits the fibrillization of PTH_25–37_ in ratios of up to one-fifth. The approach reported here is designed to control the concentration of PTH-peptides, where the bioactive form can be catalytically controlled by an added photoswitchable peptide.

## 1. Introduction

Fibrillization of proteins and peptides is a supramolecular process [1,2] that leads to the formation of peptide aggregates, containing a cross-β-sheet motif [3]. It involves multiple steps [4] and is associated with many diseases such as Alzheimer’s disease, Parkinson’s disease or diabetes type II [5,6,7]. However, in the past decades, it has also been associated with amyloids with distinct physiological functions, so-called functional amyloids, which are found in lower organisms [8,9,10,11]. Subsequently, functional amyloids were also discovered in humans, whereby the amyloid can be the active physiological form [12,13] or the storage form of peptide hormones [14].

The parathyroid hormone, abbreviated PTH, is a human hormone secreted by the parathyroid glands [15], with PTH-like peptides also known from other animals [16,17]. It is expressed as a 115 residue pre-pro-protein, whereby the first 25 amino acids at the *N*-terminus (referred to PTH_−31–−7_) serve as a signaling peptide for the transport to the endoplasmic reticulum and are removed by a signal peptidase [18]. The formed pro-peptide is subsequently transferred to the Golgi apparatus and the *N*-terminal six amino acids (referred to PTH_−6–−1)_ are proteolytically removed [19]. Before mature PTH_1–84_ is released into the blood, it is stored in secretory granules as amyloid fibrils [20]. The physiological role is well studied [21,22], being important in the maintenance of extracellular fluid calcium and phosphorus homeostasis. The receptor is mainly activated through the first 34 *N*-terminal amino acids [23], wherefore recombinant PTH_1–84_ and recombinant PTH_1–34_ are approved drugs against osteoporosis, Natpara^®^ and Forteo^®^, respectively. However, its fibrillization has barely been investigated. Thus far, it is known that the amyloid fibrils of PTH_1–84_ are formed by the amino acid residues R25-L37, and the thermodynamic stability of the fibrils is sufficiently low to dissociate after dilution [20]. Thus, control over the fibrillization of amyloids and PTH specifically represents an important approach for controlling its factual concentration for in vivo applications, placing modulators of fibrillization and thus reversible fibrillization into the focus of pharmaceutically applicable proteins [24,25,26,27].

**Figure 1:**
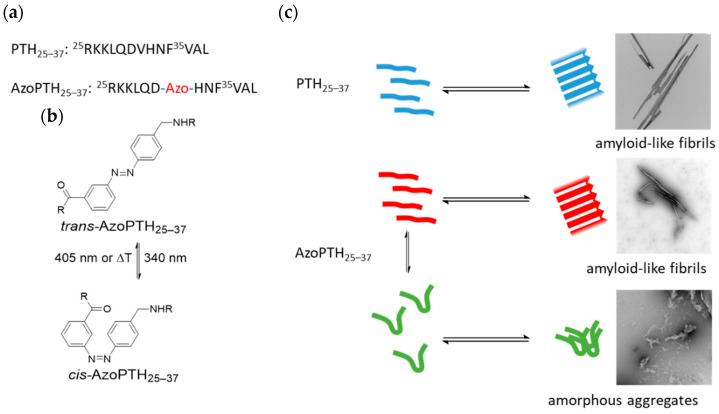
(**a**) Primary sequence of PTH_25–37_ and the azobenzene-modified PTH_25–37_ (AzoPTH_25–37_, azobenzene-moiety highlighted in red). (**b**) *Cis*-*trans*-isomerization of the incorporated 3,4′-AMPB switch. (**c**) Equilibrium of the monomeric peptides PTH_25–37_ and AzoPTH_25–37_ in both forms and their aggregates.

In the past decades, the photoinduced switching of protein functionalities has emerged as an important concept to modulate protein function, often by modulations in binding specificity between proteins and ligands. Thus, not only enzymes have been equipped with photosensitive switches, but also larger protein complexes, involved in many physiological or neurological functions [28]. To this end, artificial photoswitches are embedded into either the main chain or side chains of polypeptides, in order to change their secondary structures by photoinduced conformational changes of the photoswitches. Thereby, a plethora of different photoswitches, such as those based on cis-trans-isomerization of azo-dyes [29,30] stilbenes [31] and hemithioindigos [32,33], have been developed. Important for the proper use of a specific photoswitch inside a polypeptide chain is not only the quest to retain the initial (functional) secondary structure of the protein, but also to achieve a reasonably stable conformation after photoswitching, so as to allow for sufficient time to exert the desired effect. Many examples of such sufficiently stable and also reversible photoswitches have been reported, allowing one to modulate several expects of protein function [34,35,36,37,38,39]. Here, we report on an approach to modulate the fibrillization of PTH, equipped with a photoswitch at a specific position in the peptide sequence, in order to reversibly trigger its aggregation/disaggregation (see Figure 1).

In view of the functional design of the modified PTH_25–37_, we sought to embed the photoswitch into a region of the protein where aggregation is still possible, but only in a specific (untriggered) conformation of the photoswitch, whereby fibrillization should be inhibited after the conformational change. As a model system, we chose peptides derived from the PTH fibril core structure, including the amino acids 25R-37L (Figure 1a) [20], which is able to form fibrils itself. In addition, we investigated the influence of both conformations on the fibrillization of the unmodified peptide. As the photoswitch we chose a structural motif from the class of azobenzenes, as they are well known for enabling reversible control of peptide conformation [29,34,39,40,41]. Specifically we chose the azobenzene derivate 3-{[(4-aminomethyl)phenyl]diazenyl}benzoic acid (3,4′-AMPB; Figure 1b) [42], which is known to introduce a significant geometric change. 3,4′-AMPB displays both: a high photoisomerization yield and a sufficient thermodynamically stability of the *cis*-isomer [41]. If desired, the photoswitch can be reversed via irradiation at 405 nm, or thermally, with a half-life time of more than 20 h in the dark. We hypothesized that the incorporation of the azobenzene into the backbone would allow us to switch between the *cis*- and the *trans*-conformation, whereby one of them is able to fibrillize and the other one is not. Furthermore, azobenzenes in their *cis*-conformation are known to mimic β-hairpins, which allowed us to investigate the hypothesis if the PTH fibrils possess a turn region like amyloid fibrils from other peptides [43,44,45].

## 2. Materials and Methods

### 2.1. General

All technical solvents were distilled prior to use. Air- and moisture-sensitive reactions were carried out in flame-dried glassware under atmospheric pressure of nitrogen. 2-(6-Chloro-1-H-benzotriazole-1-yl)-1,1,3,3-tetramethylaminium hexafluorophosphate (HCTU), *N*-methyl-morpholine (NMM), *N*,*N*-dicyclohexylcarbodiimide (DIC), *N*-Hydroxybenzotriazole (HOBT), trifluoroacetic acid, 4-aminobenzylamine, and oxone^®^ were purchased from Sigma Aldrich (Taufkirchen, Germany). 9-Fluorenylmethyl-*N*-succinimidylcarbonat (Fmoc-OSu) was received from Fluorochem. 3-Aminobenzoic acid was purchased from Merck (Darmstadt, Germany). All these chemicals were used without further purification.

NMR spectra were recorded on a Varian Gemini 400 or 500 spectrometer (400 MHz or 500 MHz; Agilent Technologies, Waldbronn, Germany) at 27 °C in DMSO--*d*_6_ (99.8 Atom%D; Chemotrade, Düsseldorf, Germany) or D_2_O (99.8 Atom%D; Sigma-Aldrich, Taufkirchen, Germany). Chemical shifts are given in ppm and referred to the solvent residual signal (DMSO--*d_6_*: δ = 2.50 ppm and δ = 39.5 ppm; D_2_O: δ = 4.79 ppm). The following abbreviations were used for ^1^H- and ^13^C-NMR peaks’ assignment: s = singlet, d = doublet, t = triplet, td = triplet of doublet, and m = multiplet. MestReNova (version 6.0.2–5475, Mestrelab Research S.L., Santiago de Compostela, Spain) was used for data interpretation.

ESI-ToF mass spectrometry was performed on a Bruker Daltonics microTOF (Bruker Corporation, Billerica, MA, USA). Samples were dissolved in HPLC-grade solvents (MeOH, THF, or mixtures; Sigma Aldrich, Taufkirchen, Germany) at concentrations of 0.1 mg/mL and measured via direct injection with a flow rate of 180 μL/h using the positive mode with a capillary voltage of 4.5 kV. The spectra were analyzed with otofControl (version 3.4, Bruker Daltonik, Bremen, Germany).

### 2.2. Organic Synthesis

Fmoc-protected 3,4′-AMPB was synthesized in two steps according to literature procedures [42,46].

### 2.3. Peptide Synthesis and Purification

Solid-phase peptide synthesis was utilized on an automated peptide synthesizer MultiPep RS (Intavis AG, Koeln, Germany) using standard Fmoc-chemistry and preloaded resins. Standard coupling of all protected natural amino acids was performed as single couplings in dimethylformamid (DMF) using 5 equivalents of amino acids, HCTU as coupling reagents, and 10 equivalents of NMM as base for 1 h at room temperature. Special building groups, such as Fmoc-3,4′-AMPB, were coupled with 3 equivalents using DIC and HOBT in DMF/*N*-methyl-2-pyrrolidone (NMP) at room temperature and with gentle shaking in the dark overnight.

The *N*-terminal Fmoc-protecting group was removed by washing the resin with 20% piperidine for 20 min. The final side chain deprotection and cleavage from the resin employed a mixture of trifluoroacetic acid and water (90:10 Vol%) with gentle agitation for 2 h at room temperature.

The crude peptides were purified to >95% purity using preparative RP-HPLC (Gilson, Limburg, Germany). For both analytical and preparative use, the mobile phase was a mixture of water (eluent A) and acetonitrile (eluent B), respectively, each containing 0.1% trifluoroacetic acid. Samples were eluted with a linear gradient from 5% B to 95% B in 15 min for analytical runs and in 90 min for preparative runs on a semipreparative PLRP-S column (300 × 25 mm, 8 μm; Agilent Technologies, Waldbronn, Germany). Finally, all peptides were characterized by analytical HPLC Dionex Ultimate 3000 (Thermo Fisher Scientific, Dreieich, Germany) using a PLRP-S column (150 × 4.6 mm, 3 μm; Agilent Technologies, Waldbronn, Germany) and MALDI-MS (Bruker Microflex LT, Bremen, Germany), which gave the expected [M+H]^+^ mass peaks.

### 2.4. Azobenzene Peptide Photoisomerization

*Trans* → *cis* isomerization was performed by irradiating the dissolved peptide in a 1 cm quartz cuvette for 30 min with light of 340 nm wavelength using a 50 W mercury lamp (VEB) and a 340 nm band pass filter (FB340-10, Thorlabs, Bergkirchen, Germany) under stirring. For *cis* → *trans* isomerization, the dissolved peptide was irradiated with light of 405 nm wavelength using a 1.4 W LED (M405L4, Thorlabs, Bergkirchen, Germany) for 30 min under stirring.

### 2.5. Aggregation Kinetics

ThT-monitored fibrillization assays of artificial peptides and mixtures with PTH_25–37_ were investigated by fluorescence intensity measurements using thioflavin T (ThT) as fluorescent dye. Lyophilized peptides were dissolved in 50 mM Na_2_HPO_4_ buffer solution with a pH value of 7.4 in a concentration of 2 mg/mL and kept on ice for the next steps. The samples were centrifuged at 13,000× *g* rpm for 10 s and the concentrations were determined with a JASCO V-660 absorbance spectrometer (JASCO, Pfungstadt, Germany; PTH_25–37_ by absorbance at 205 nm and the molar extinction coefficient of 49,310 cm^−1^M^−1^; *trans*-AzoPTH_25–37_ by absorbance at 327 nm and the molar extinction coefficient of 13,000 cm^−1^M^−1^). *Cis*-AzoPTH_25–37_ was produced as described before. The solutions were centrifuged at 10,000 rpm for 1 h at 4 °C, the supernatant was transferred to another tube. The protein solutions were mixed in the desired ration and diluted with 50 mM Na_2_HPO_4_ buffer (pH 7.4) to obtain final concentrations of 0/100 μM PTH_25–37_, 50 μM ThT, and 0/10/20/50/100 μM AzoPTH_25–37_. For each sample, a total volume of 480 μL was prepared and 3 × 150 μL were transferred to a medium binding 96-well plate (Greiner Bio-One, Kremsmünster, Austria). The plate was sealed with a microplate cover. The fluorescence intensity was monitored at 37 °C using a BMG FLUOStar Omega multi-mode plate reader (BMG LABTECH, Ortenberg, Germany) using fluorescence excitation and emission wavelengths at 460 nm and 485 nm, respectively. One measurement cycle of 5 min consisted of double-orbital shaking for 150 s and incubating for 150 s.

### 2.6. Transmission Electron Microscopy (TEM)

TEM images were taken with an electron microscope (EM 900; Zeiss, Oberkochen, Germany) at 80 kV acceleration voltage. For preparation, 5 μL of the peptide solution were added on Formvar/Cu grids (mesh 200). After 3 min of incubation, the grids were gently cleaned with water for o1 min and then negatively stained using uranyl acetate (1%, *w*/*v*) for 1 min.

### 2.7. Seeding Assay

The seeding assay follows the same procedure as the ThT-monitored fibrillization assay for the determination of the aggregation kinetics. In addition, the final samples contained 20 μM of seeds from *trans*-AzoPTH_25–37_ fibrils. The seeds were prepared via ultrasonification of a 100 μM mature *trans*-AzoPTH_25–37_ fibrils solution (Sonifier W-250 D, Branson Ultraschall, Dietzenbach, Germany; 15 times, 1 s 10% amplitude, 1 s pause).

## 3. Results & Discussion

### 3.1. Chemistry 

**Figure 2:**
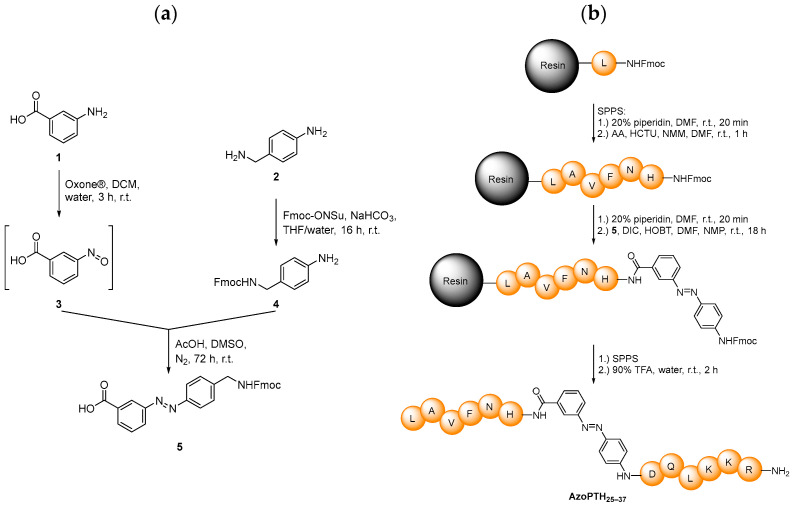
(**a**) Synthesis of Fmoc-protected *trans*-3,4′-AMPB **5**. (**b**) Solid-phase peptide synthesis strategy towards the peptide AzoPTH_25–37_.

To investigate the fibrillization behavior of PTH_25–37_, the azobenzene switch was incorporated directly into the peptide backbone. We selected the 3,4′-azobenzene motif (Figure 1b) [42]. As it possesses suitable photochemical properties, e.g., an excellent half-life time with a stability larger than 20 h and switching wavelengths >300 nm. These are easily addressable by our photophysical equipment and also avoid eventual photodegradation. The synthesis was conducted in two steps (Figure 2a): in the first step, we conducted the Fmoc-protection of **2** [46], which in the second step reacts in a Mills reaction with an in situ-generated nitroso compound **3** to obtain the Fmoc-protected 3,4′-AMPB **5** in an overall yield of 68%.

The modified azobenzene switch **5**, bearing the proper functionalities for Fmoc-chemistry, was incorporated into the peptide backbone of PTH_25–37_ via solid-phase peptide synthesis (Figure 2b). It replaces V31 in the artificial peptide AzoPTH_25–37_, due to its central position along the peptide, expecting the largest impact on fibrillization after photoswitching. Furthermore, we probed the replacement of D30 or the insertion between D30 and V31, which led to a greater loss of solubility in the fibrillization buffer (240 μM vs. 25 μM vs. 60 μM; Table S1). Thus, several of the generated peptides displayed strongly reduced solubility—an effect that is important for the subsequent investigations. All peptides were obtained in yields of 10–19%, and high purities as proven by both HPLC and MALDI-ToF measurements, in addition to 500 MHz NMR spectroscopy (Figures S1–S5 and S13–S15).

### 3.2. Photophysical Properties

**Figure 3:**
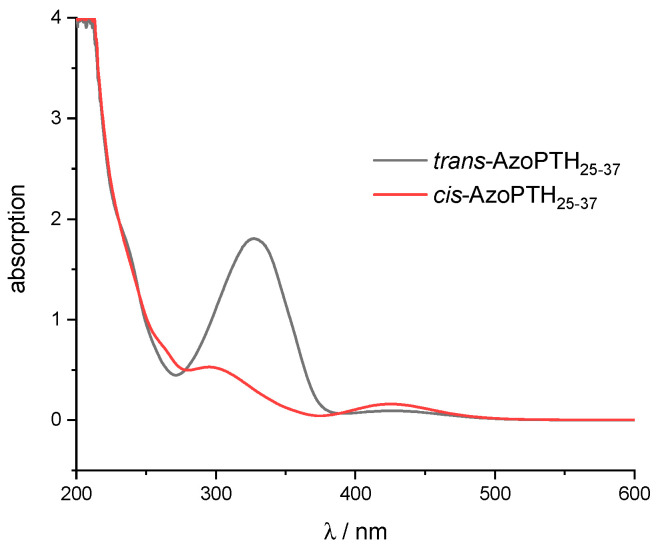
UV/Vis absorption spectra for *trans*-AzoPTH_25–37_ after synthesis and for the *cis*-enriched photo-stationary state after irradiation at 340 nm, which almost corresponds to *cis*-AzoPTH_25–37_.

We first studied the photophysical properties of the *cis*-*trans*-isomerization of AzoPTH_25–37_ (Figure 1b) by UV/Vis spectroscopy and HPLC analysis in pure water in order to minimize effects of a potential self-assembly and to quantify the generated amounts of the respective *cis*/*trans*-modified peptides before and after photoswitching. The UV/Vis spectra for the pure isomers (Figure S6) were separated from the spectra of *trans*-enriched AzoPTH_25–37_ in the thermodynamically stable state after synthesis and in the *cis*-enriched photostationary state (PSS, Figure 3) with Wolfram Mathematica 12.2. The *trans*-isomer displays an absorption maximum at 327 nm (ε = 13,000 cm^−1^M^−1^) and a second maximum at 427 nm, while the *cis*-isomer possesses maxima at 288 nm and 433 nm. Both isomers display two isobestic points at 278 nm and 388 nm. They represent in the thermodynamically stable state a *cis*-*trans* ratio of 3:97. Under irradiation with UV light (340 nm), the *cis*-content could be increased of up to 82% in the *cis*-enriched PSS. Visible light (405 nm) yields 76% of the *trans*-isomer in the *trans*-enriched PSS via the back reaction. The difference of the *trans*-content between the *trans*-enriched PSS at 405 nm and the thermodynamically stable state arises from the overlapping of the n → π* transitions of both isomers at this wavelength [47]. The rate of thermal *cis*-to-*trans* isomerization of AzoPTH_25–37_ follows first-order kinetics, and was determined by monitoring the increase of the π → π* absorption band at 327 nm (Figure S7) via time-dependent UV measurements. In the absence of light at 37 °C, *cis*-AzoPTH_25–37_ isomerizes thermally with a rate constant of 3.53 × 10^−6^ s^−1^, corresponding to a half-life time of 79 h.

### 3.3. Aggregation Kinetics and TEM-Recordings

In order to determine the kinetics of fibril formation of both modified AzoPTH_25–37_ isomers a thioflavin T (ThT)-monitored fibrillization assay was conducted and compared to PTH_25–37_. ThT is a benzothiazole compound that binds to the cross-β-sheet structure of amyloid fibrils [48]. Causing a large red shift of fluorescence excitation of ThT, which in turn enables the selective excitation of amyloid fibril-bound ThT and therefore the in situ observation of fibril formation.

**Figure 4:**
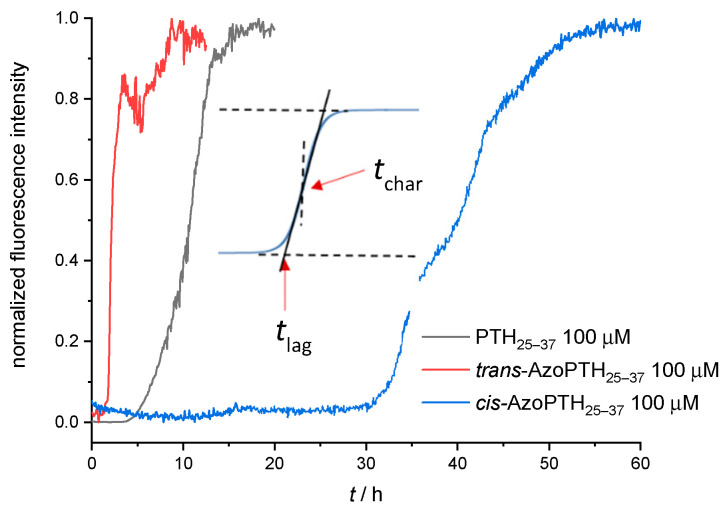
ThT-monitored fibrillization assay of PTH_25–37_, *cis*-AzoPTH_25–37_, and *trans*-AzoPTH_25–37_ (average of triplets; *T* = 37 °C, buffer = 50 mM Na_2_HPO_4,_ pH = 7.4): (black) PTH_25–37_ (100 μM), (red) *trans*-AzoPTH_25–37_ (100 μM), and (blue) *cis*-AzoPTH_25–37_ (100 μM).

In a first attempt, the fibrillization kinetics for pure *trans*-AzoPTH_25–37_, *cis*-AzoPTH_25–37_, and the PTH-derived peptide PTH_25–37_ were measured at 37 °C and the results are shown in Figure 4. Two characteristic times were used to characterize the fibrillization (Figure 4, Table 1): the lag time *t*_lag_ corresponds to the time before an increase in the fluorescence signal occurs; the characteristic time *t*_char_ indicates at which time 50% of the maximum fluorescence was reached.

The self-assembly of the *trans*-AzoPTH_25–37_ was accelerated compared to PTH_25–37_, while *cis*-AzoPTH_25–37_ exhibited the opposite effect (Figure 4). The first increase of ThT fluorescence was observable after >30 h. Furthermore, *cis*-AzoPTH_25–37_ shows a biphasic fibrillization behavior, while *trans*-AzoPTH_25–37_ and PTH_25–37_ show monophasic fibrillization. Compared to PTH_25–37_, the magnitude of the ThT fluorescence of both AzoPTH_25–37_ isomers was significant lower (Figure S9). This effect might arise from fluorescence quenching via the azobenzene moiety. To test this hypothesis, the fluorescence lifetime of ThT was measured either alone, in the presence of PTH_25–37_ fibrils, or in the presence of *trans*-AzoPTH_25–37_ fibrils (Figure S8). As expected the lifetime is increased in the presence of PTH_25–37_ fibrils compared to the control experiment, while it is decreased significantly in the presence of *trans*-AzoPTH_25–37_, which further supports our concept. In addition, this effect could be enhanced from a reduced binding affinity of ThT through a different peptide conformation of the fibril.

**Figure 5:**
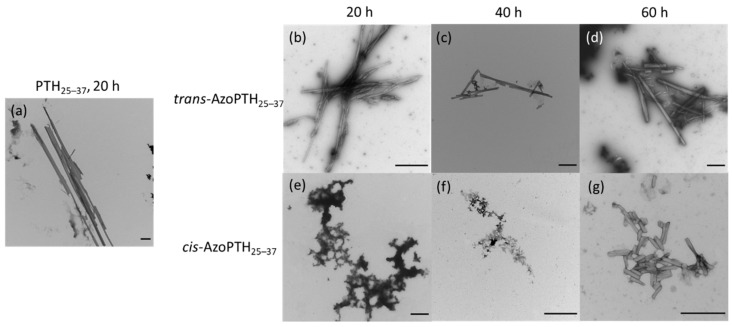
TEM recordings of fibrils obtained from PTH_25–37_, *cis*-AzoPTH_25–37_, and *trans*-AzoPTH_25–37_ at (*T* = 37 °C, buffer = 50 mM Na_2_HPO_4,_ pH = 7.4) after different time points (all scale bars corresponds to 500 nm). (**a**). PTH_25–37_ after 20 h, (**b**) *trans*-AzoPTH_25–37_ after 20 h (100 μM), (**c**) *trans*-AzoPTH_25–37_ after 40 h (100 μM), (**d**) *trans*-AzoPTH_25–37_ after 60 h (100 μM), (**e**) *cis*-AzoPTH_25–37_ after 20 h (100 μM), (**f**) *cis*-AzoPTH_25–37_ after 40 h (100 μM), and (**g**) *cis*-AzoPTH_25–37_ after 60 h (100 μM).

The observations of the ThT-monitored fibrillization assay were supported by negative stain transmission electron microscopy (TEM) after different time points (Figure 5). After 20 h, amyloid fibrils were only observable for PTH_25–37_ and *trans*-AzoPTH_25–37_ (Figure 5a,b), while *cis*-AzoPTH_25–37_ formed amorphous aggregates (Figure 5e). Both peptides produced straight fibrils, whereby the single fibrils of PTH_25–37_ were larger (>6 μm vs. <1.5 μm) and tend to aggregate further. Interestingly, we found fibrils after 60 h for *cis*-AzoPTH_25–37_ (Figure 5g), which matched in the morphology those of *trans*-AzoPTH_25–37_ even if they were significantly shorter (<300 nm). This may result from the thermal *cis*-*trans*-isomerization, as the *cis*-content decreases and is reduced to 48% after 60 h.

**Figure 6:**
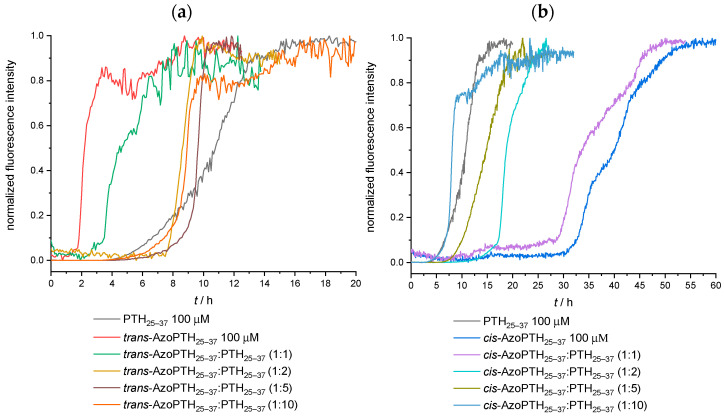
(**a**) ThT-monitored fibrillization assay of PTH_25–37_ and mixtures with *trans*-AzoPTH_25–37_ (average of triplets; *T* = 37 °C, buffer = 50 mM Na_2_HPO_4,_ pH = 7.4): (black) PTH_25–37_ (100 μM), (red) *trans*-AzoPTH_25–37_ (100 μM), (green) *trans*-AzoPTH_25–37_:PTH_25–37_ (100 μM:100 μM), (dark yellow) *trans*-AzoPTH_25–37_:PTH_25–37_ (50 μM:100 μM), (brown) *trans*-AzoPTH_25–37_:PTH_25–37_ (20 μM:100 μM), and (orange) *trans*-AzoPTH_25–37_:PTH_25–37_ (10 μM:100 μM) (**b**) ThT-monitored fibrillization assay of PTH_25–37_ and mixtures with *cis*-AzoPTH_25–37_ at 37 °C: (black) PTH_25–37_ (100 μM), (blue) *cis*-AzoPTH_25–37_ (100 μM), (purple) *cis*-AzoPTH_25–37_:PTH_25–37_ (100 μM:100 μM), (cyan) *cis*-AzoPTH_25–37_:PTH_25–37_ (50 μM:100 μM), (olive) *cis*-AzoPTH_25–37_:PTH_25–37_ (20 μM:100 μM), and (light blue) *cis*-AzoPTH_25–37_:PTH_25–37_ (10 μM:100 μM).

In further experiments, we investigated the (catalytic) influence of the AzoPTH_25–37_ isomers on the fibrillization of PTH_25–37_ (Figure 6). We previously observed such catalytic effects of β-turn modified amyloids (Aβ) on the fibrillization of the Alzheimer peptide Aβ_1–40_ [49]. Thus 100 μM of PTH_25–37_ were fibrillized in the presence of various concentrations of the respective AzoPTH_25–37_ isomer (10/20/50/100 μM). Kinetic measurements revealed that the fibrillization behavior of PTH_25–37_ was affected in the same way as the pure AzoPTH_25–37_ isomers. 

While *trans*-AzoPTH_25–37_ accelerated the fibrillization and therefore reduced *t*_lag_ and *t*_char_ of the mixtures (Figure 6a), *cis*-AzoPTH_25–37_ inhibited the fibrillization and extended *t*_lag_ and *t*_char_ (Figure 6b). Interestingly, the biphasic fibrillization behavior of *cis*-AzoPTH_25–37_ was also observable for the *cis*-AzoPTH_25–37_:PTH_25–37_ (100 μM:100 μM) mixture. These effects are reduced with decreasing concentration of the respective AzoPTH_25–37_ isomer. While the mixtures with *trans*-AzoPTH_25–37_ exhibited a concentration below 50 μM, *trans*-AzoPTH_25–37_ had a higher *t*_lag_ than pure PTH_25–37_. However, *t*_char_ was still shorter, and the stationary phase of the fibrillization was reached earlier.

**Figure 7:**
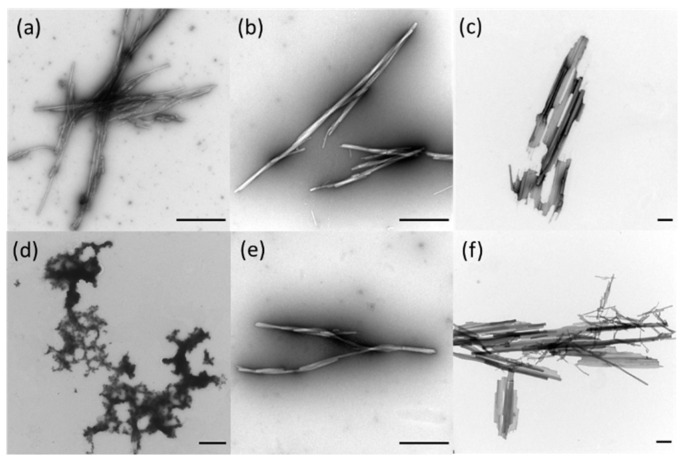
TEM recordings of fibrils obtained from PTH_25–37_, *cis*-AzoPTH_25–37_, and *trans*-AzoPTH_25–37_ at 37 °C after 20 h (scale bar = 500 nm): (**a**) *trans*-AzoPTH_25–37_ (100 μM), (**b**) *trans*-AzoPTH_25–37_:PTH_25–37_ (100 μM:100 μM), (**c**) *trans*-AzoPTH_25–37_:PTH_25–37_ (10 μM:100 μM), (**d**) *cis*-AzoPTH_25–37_ (100 μM), (**e**) *cis*-AzoPTH_25–37_:PTH_25–37_ (100 μM:100 μM), and (**f**) *cis*-AzoPTH_25–37_:PTH_25–37_ (10 μM:100 μM).

TEM images were recorded for the peptide mixtures after 20 h (Figure 7). In contrast to the pure peptides, we could observe fibrils for all investigated ratios. Interestingly, the fibrils formed by the mixtures exhibit a similar twisted morphology regardless of the used AzoPTH_25–37_ isomer. Furthermore, the formation of larger aggregates like for the pure PTH_25–37_ (Figure 5) were only observed for a ratio of 1:10, indicating that the AzoPTH_25–37_ inhibits the formation of larger fibril aggregates.

### 3.4. Seeding Experiments

**Figure 8:**
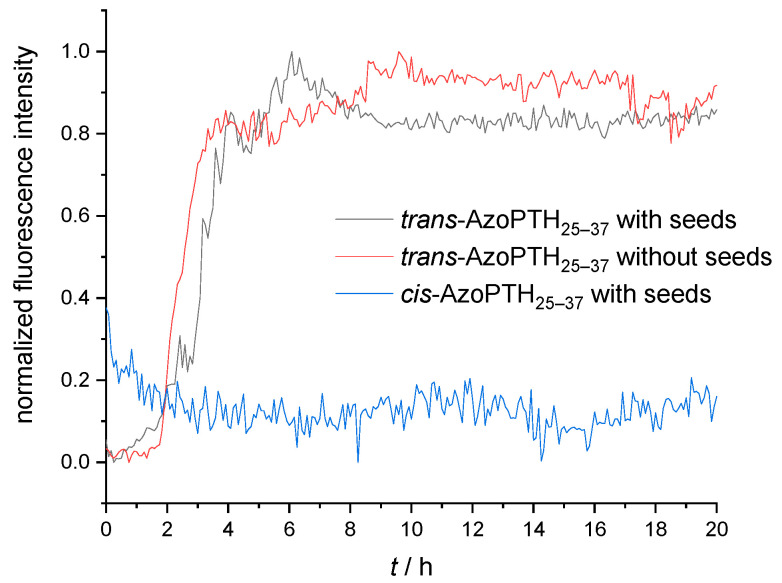
ThT-monitored fibrillization assay of cross-seeding studies with *cis*-AzoPTH_25–37_ and *trans*-AzoPTH_25–37_ monomeric peptides and mature *trans*-AzoPTH_25–37_ fibrils as seeds (average of triplets; c_monomer_ = 100 μM, c_seed_ = 20 μM, *T* = 37 °C, buffer 50 mM Na_2_HPO_4_, pH = 7.4): (black) *trans*-AzoPTH_25–37_ with seeds, (red) *trans*-AzoPTH_25–37_ without seeds, and (blue) *cis*-AzoPTH_25–37_ with seeds.

To determine whether both isomers of AzoPTH_25–37_ are able to form fibrils or only the *trans*-isomer, we investigated, if *trans*-AzoPTH_25–37_ fibrils were able to induce seeding [50]. A 100 μM solution of each isomer was treated with 20 μM of mature *trans*-AzoPTH_25–37_ fibrils, and the kinetics of the fibril formation were investigated via a ThT-monitored fibrillization assay (Figure 8). While the fibrillization of the *trans*-isomer was accelerated compared to the unseeded monomer, we were not able to observe fibrillization for the *cis*-isomer. This indicates that the *cis*-isomer is unable to nucleate amyloid formation as well as elongate preformed fibrils. The observed fibrils after 60 h for the *cis*-isomer are presumably formed by the thermally isomerized *trans*-isomer.

## 4. Conclusions

We here report for the first time a photoswitchable fibrillizing PTH-derived peptide, which is able to modulate its fibrillization by embedding an azobenzene photoswitch in the middle of PTH_25–37_. PTH_1–84_ is a peptide hormone, which is stored as functional amyloids in secretory granules. Its physiological role is well studied, but it still lacks detailed information about its exact fibril structure. We used the 3,4′-AMPB photoswitch to investigate the fibril formation of the fibril core fragment of PTH_1–84_ by incorporating the azobenzene into the peptide backbone, yielding the modified PTH-derived peptide AzoPTH_25–37_. We could show that the *trans*-isomer is able to form fibrils, while the *cis*-isomer induces a conformational change that inhibits fibril formation. Hypothetically, we can also conclude that there might not be a β-turn in the fibril structure of PTH_1–84_, as the *cis*-conformer would be reminiscent of such a structure, whereas the *trans*-conformer would not. Most importantly, we were able to show that the modified peptides can catalytically inhibit fibrillization of the PTH_25–37_, underscoring the importance of seeding during this fibrillization process, which in the future allows for a reversible triggering of the fibrillization by light as an external stimulus. Studies are in progress to investigate if the photocontrol is also possible with the photoswitch at other positions of the backbone and if we can also control the fibrillization of full-length PTH_1–84_ with ours or other modified peptides. This represents a novel strategy to control bioavailability of proteins, specifically of PTH peptides and other fibrillating peptides, where not only the concentration of the bioactive form can be controlled by an added photoswitchable peptide, but also the fibrillization as such, important to guide nerve cell regeneration and other directed growth processes in euraryotic cells. For a potential clinical perspective, we want to investigate the cytotoxicity of our peptides as well as the ability to influence the fibrillization of larger PTH-derived peptides (e.g., PTH_1–34_ and PTH_1–84_) in vitro and in vivo. As known from other azobenzene containing drugs/prodrugs (e.g., Prontosil), the azobenzene moiety is metabolized in liver tissue via azoreductases, yielding two aniline moieties or through intestinal microbes [51,52]. This is potentially important for the photoswitching inside cells by light, allowing them to tune the reversible fibrillization of other amyloidogenic peptides, which important for regeneration of nerve cells, as reported earlier. Thus, peptide fibrils can seed potential harmful amyloidogenic peptides, which is known from recent work quite prominently [53]. This is a strategy to trigger fiber-formation from the outside via photochemical triggering—thus avoiding the toxic effects of the fibers outside the cells but enabling triggered fibrillization inside the cell to exert the desired effects, allowing them to promote the recovery of spinal cord injuries.

## Figures and Tables

**Table 1:** Fibrillization parameters (*t*_lag_, *t*_char_) of PTH_25–37_, *cis*-AzoPTH_25–37_, *trans*-AzoPTH_25–37_, and mixtures thereof (*T* = 37 °C, buffer = 50 mM Na_2_HPO_4,_ pH = 7.4).

Sample	*t*_lag_ [h]	*t*_char_ [h]
PTH_25–37_ (100 μM)	7.2	10.9
*cis*-AzoPTH_25–37_ (100 μM)	34.4	42.4
*cis*-AzoPTH_25–37_:PTH_25–37_ (100 μM:100 μM)	27.9	35.7
*cis*-AzoPTH_25–37_:PTH_25–37_ (50 μM:100 μM)	16.3	21.2
*cis*-AzoPTH_25–37_:PTH_25–37_ (20 μM:100 μM)	10.1	14.5
*cis*-AzoPTH_25–37_:PTH_25–37_ (10 μM:100 μM)	6.9	7.9
*trans*-AzoPTH_25–37_ (100 μM)	1.6	2.1
*trans*-AzoPTH_25–37_:PTH_25–37_ (100 μM:100 μM)	3.0	4.8
*trans*-AzoPTH_25–37_:PTH_25–37_ (50 μM:100 μM)	8.0	8.6
*trans*-AzoPTH_25–37_:PTH_25–37_ (20 μM:100 μM)	8.7	9.7
*trans*-AzoPTH_25–37_:PTH_25–37_ (10 μM:100 μM)	7.5	8.9

## Data Availability

Data are contained within the article.

## Supplementary Materials

The following supporting information can be downloaded at: https://www.mdpi.com/article/10.3390/biomedicines10071512/s1, Figure S1: (A) HPLC-trace of AzoPTH_25–37_ (*cis*-isomer at 5.353, *trans*-isomer at 5.530). (B) MALDI-spectrum of AzoPTH_25–37_; Figure S2: (A) HPLC-trace of SP1 (*cis*-isomer at 5.557, *trans*-isomer at 5.790). (B) MALDI-spectrum of SP1; Figure S3: (A) HPLC-trace of SP2 (*cis*-isomer at 5.223, *trans*-isomer at 5.460). (B) MALDI-spectrum of SP2; Figure S4: (A) HPLC-trace of SP3 (*cis*-isomer at 5.560, *trans*-isomer at 5.807). (B) MALDI-spectrum of SP3; Figure S5: (A) HPLC-trace of SP4 (*cis*-isomer at 5.363, *trans*-isomer at 5.547). (B) MALDI-spectrum of SP4; Figure S6: Separated UV/Vis-spectra of the pure isomers of AzoPTH_25–37_; spectra were seperated with Wolfram Mathematica 12.2; Figure S7: (A) UV/Vis-spectra of *trans*-isomer, *cis*-enriched PSS, and *cis*-enriched PSS sample after distinct time points in the dark. (B) logarithmic application of the absorption change over time to determine rate constant *k* and half-life time *t*_1/2_; Figure S8: Time-resolved fluorescence measurement (excitation wavelength = 460 nm, emission wavelength = 480 nm) of unbound ThT (black), ThT bound to PTH_25–37_ fibrils (dark green), ThT bound to *trans*-AzoPTH_25–37_ fibrils (light green); Figure S9: ThT monitored fibrillation assays (c = 100 μM, 37 °C, 50 mM Na_2_HPO_4_, pH 7.4). (A) PTH_25–37_, (B) *trans*-AzoPTH_25–37_, (C) *cis*-AzoPTH_25–37_; Figure S10: ThT monitored fibrillization assays of mixtures of PTH_25–37_, *trans*-AzoPTH_25–37_, and *cis*-AzoPTH_25–37_ (37 °C, 50 mM Na_2_HPO_4_, pH 7.4). (A) *trans*-AzoPTH_25–37_:PTH_25–37_ (100 μM:100 μM), (B) *cis*-AzoPTH_25–37_:PTH_25–37_ (100 μM:100 μM), (C) *trans*-AzoPTH_25–37_:PTH_25–37_ (50 μM:100 μM), (D) *cis*-AzoPTH_25–37_:PTH_25–37_ (50 μM:100 μM), (E) *trans*-AzoPTH_25–37_:PTH_25–37_ (20 μM:100 μM), (F) *cis*-AzoPTH_25–37_:PTH_25–37_ (20 μM:100 μM), (G) *trans*-AzoPTH_25–37_:PTH_25–37_ (10 μM:100 μM), (H) *cis*-AzoPTH_25–37_:PTH_25–37_ (10 μM:100 μM); Figure S11: ^1^H-NMR spectrum (top; 400 MHz, DMSO-*d*_6_) and ^13^C-NMR spectrum (bottom; 100 MHz, DMSO-*d*_6_) of (9*H*-Fluoren-9-yl)methyl (4-aminobenzyl)carbamate; Figure S12: ^1^H-NMR spectrum (top; 400 MHz, DMSO-*d*_6_) and ^13^C-NMR spectrum (bottom; 100 MHz, DMSO-*d*_6_) of Fmoc-3,4′-AMPB (mixture of isomers); Figure S13: ^1^H-NMR spectra (500 MHz, D_2_O) of AzoPTH_25–37_ (top, *trans*-isomer) and SP1 (bottom, *trans*-isomer); Figure S14: ^1^H-NMR spectra (500 MHz, D_2_O) of SP2 (top, *trans*-isomer) and SP3 (bottom, *trans*-isomer); Figure S15: ^1^H-NMR spectrum (500 MHz, D_2_O) of SP4 (*trans*-isomer); Scheme S1: Synthesis of Fmoc-protected 3,4′-AMPB 7. (a) Fmoc-ONSu, triethylamin, DMF/MeCN, 16 h, room temperature. (b) Oxone^®^, DCM, water, 3 h, room temperature. (c) AcOH, DMSO, N2, 72 h, room temperature; Table S1: Primary sequence and solubility in 50 mM Na_2_HPO_4_ buffer (pH 7.4) of peptides AzoPTH_25–37_ and SP1–SP4.
