# Supplementary material for: Modulating the Fibrillization of Parathyroid-Hormone (PTH) Peptides: Azo-Switches as Reversible and Catalytic Entities
Source: Biomedicines. 2022 Jun 26;10(7):1512. doi: 10.3390/biomedicines10071512 (PMC9313110; doi:10.3390/biomedicines10071512)
Supplement: Supplementary file 1 [file biomedicines-10-01512-s001.zip › biomedicines-1761126-supplementary.pdf]

Modulating the fibrillization of parathyroid-hormone (PTH) peptides: azo-switches as reversible and catalytic entities.

Authors: André Paschold, Bruno Voigt, Gerd Hause, Tim Kohlmann, Sven Rothemund, and Wolfgang H. Binder

Table of content

1. Synthesis of Fmoc-3,4'-AMPB ... 2

1.1. Synthesis of (9*H*-Fluoren-9-yl)methyl (4-aminobenzyl)carbamate 2 ... 2

1.2. Synthesis of Fmoc-3,4'-AMPB 5 ... 2

2. Peptide Characterisation ... 4

2.1. Peptide sequences ... 4

2.2. HPLC- and MALDI-ToF-data ... 4

2.3. Photophysical properties of $AzoPTH_{25-37}$ ... 9

2.4. Aggregation Kinetics of PTH_{25-37}, *trans*-$AzoPTH_{25-37}$, *cis*-$AzoPTH_{25-37}$, and mixtures ... 10

3. NMR-Spectra ... 12

1. Synthesis of Fmoc-3,4’-AMPB

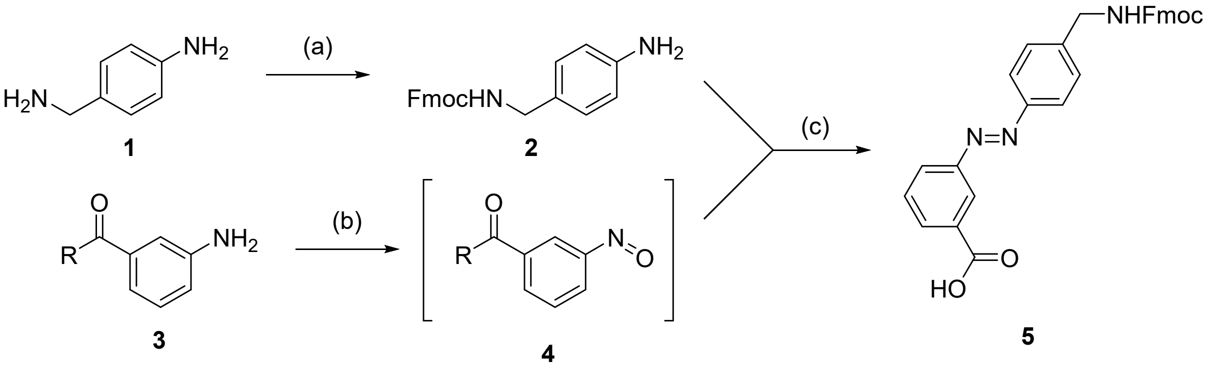

Scheme S1: Synthesis of Fmoc-protected 3,4‘-AMPB **7**. (a) Fmoc-ONSu, triethylamin, DMF/MeCN, 16 h, room temperature. (b) Oxone®, DCM, water, 3 h, room temperature. (c) AcOH, DMSO, N_2, 72 h, room temperature.

1.1. Synthesis of (9*H*-Fluoren-9-yl)methyl (4-aminobenzyl)carbamate 2

4-Aminobenzylamine (1.85 mL, 16.4 mmol, 1 equiv.) was dissolved in a mixture of 12 mL THF and 1.5 mL aqueous 5% $NaHCO_3$ solution. A suspension of Fmoc-*N*-hydroxysuccinimide (Fmos-OSu) (5.52 g, 16.4 mmol, 1 equiv.) in 20 mL THF was added slowly and the reaction mixture was stirred for 18 h at ambient temperature. 500 mL of water were added and the suspension was filtrated. The precipitate was dissolved in THF and dried over Na_2SO_4. The solvent was removed *in vacuo* and **2** was obtained as a white solid (5.45 g, 15.3 mmol, 96% yield).

^{1}H-NMR (400 MHz, DMSO-d_6) δ = 7.86 (d, *J* = 7.6 Hz, 2H, $C_{ar}H$), 7.82 (d, *J* =7.6 Hz, 2H, $C_{ar}H$), 7.40 (td, *J* = 7.5 Hz, 1.2 Hz, 1H, $C_{ar}H$), 7.32 (td, *J* = 7.5 Hz, 1.2 Hz, 1H, $C_{ar}H$), 6.97 (t, *J* = 6.3 Hz, 1H, N*H*), 6.88 (d, *J* = 8.2 Hz, 2H, $C_{ar}H$), 6.48 (d, *J* = 8.2 Hz, 2H, $C_{ar}H$), 6.26 (s, 2H, CH_2), 4.88 (s, 2H, NH_2), 3.93 (d, *J* = 5.9 Hz, 1H, CH_2).

^{13}C-NMR (100 MHz, DMSO-d_6) δ = 157.8 (*C*(O)N), 147.8 (C_{ar}N), 143.0 (C_{ar}), 139.8 (C_{ar}), 137.8 (C_{ar}), 141.2 (C_{ar}), 129.4 (C_{ar}H), 128.4 (C_{ar}H), 127.7 (C_{ar}), 121.1 (C_{ar}H), 120.5 (C_{ar}H), 114.1(C_{ar}H), 110.1 (CH_2), 43.9 (CH_2).

MS (ESI+): *m/z* = 383.11 $[M+K]^+$.

Analytical data match reported literature values.[1]

1.2. Synthesis of Fmoc-3,4’-AMPB 5

To a suspenison of 3-aminobenzoic acid (2.00 g, 14.6 mmol, 2 equiv.) in 36 mL DCM is a solution of Oxone® (8.80 g, 29.17 mmol, 4 equiv.) added dropwise. The reaction mixture is stirred at ambient temperature for 4 h. DCM is removed *in vacuo* and the formed precipitate is filtrated and washed with 100 mL water. The residue is dissolved in 175 mL DMSO and 175 mL of acetic acid are added. The solution is degassed for 30 min with N_2. (9*H*-Fluoren-9-yl)methyl (4-aminobenzyl)carbamate **2** (2.51 g, 7.29 mmol, 1 equiv.) is added and the mixture is stirred for 72 h at ambient temperature. 100 mL water

and 400 mL EtOAc were added and the phases were separated. The aqueous phase is washed two times with 400 mL EtOAc and the combined organic phases were washed two times with 200 mL of a saturated aqueous NaCl solution. The organic phase was dried over Na_2SO_4 and the solvent was removed *in vacuo*. The residue was purified by flash chromatography on normal phase silica gel (solvent: EtOAc + 0.1% FA) to obtain **5** as a mixture of the *E*- and the *Z*-isomer (1.51 g, 3.17 mmol, 43.5% yield).

^{1}H-NMR (400 MHz, DMSO-d_6) *E*:*Z*-ratio = 29:11, *E*-isomer: δ = 13.27 (s, 1H, C(O)O*H* 8.37 (t, *J* = 1.9 Hz, 1H, $C_{ar}H$), 8.15 – 8.08 (m, 2H, $C_{ar}H$), 7.93 (t, *J* = 6.2 Hz, 1H, $C_{ar}H$), 7.90 – 7.81 (m, 4H, $C_{ar}H$), 7.76 – 7.66 (m, 3H, $C_{ar}H$, NH_2), 7.46 – 7.48 (m, 6H, $C_{ar}H$), 4.38 (d, *J* = 6.7 Hz, 2H, CH_2), 4.28 (d, *J* = 6.0 Hz, 2H, CH_2), 4.24 (t, *J* = 6.8 Hz, 1H, C*H*), *Z*-isomer: 7.86 (d, *J* = 7.6 Hz, 2H, $C_{ar}H$), 7.79 – 7.69 (m, 2H, $C_{ar}H$), 7.66 (d, *J* = 7.5 Hz, 2H, $C_{ar}H$), 7.46 – 7.35 (m, 4H, $C_{ar}H$, C(O)N*H*), 7.28 (t, *J* = 7.5 Hz, 2H, $C_{ar}H$), 7.08 (d, *J* = 8.0 Hz, 2H, $C_{ar}H$), 6.99 (d, *J* = 7.8 Hz, 1H, $C_{ar}H$), 6.81 (d, *J* = 8.2 Hz, 2H, $C_{ar}H$), 4.36 (d, *J* = 6.6 Hz, 2H, CH_2), 4.20 (t, *J* = 6.7 Hz, 1H, C*H*), 4.09 (d, *J* = 6.2 Hz, 2H, CH_2).

^{13}C-NMR (100 MHz, DMSO-d_6) *E*-isomer: δ = 167.2 (*C*(O)OH), 156.9 (*C*(O)N), 152.4 (C_{ar}N), 151.3 (C_{ar}N), 144.5 (C_{ar}), 144.3 (C_{ar}), 141.2 (C_{ar}), 132.7 (C_{ar}H), 132.2 (C_{ar}H), 130.4 (C_{ar}), 128.4 (C_{ar}H), 128.0 (C_{ar}H), 127.8 (C_{ar}H), 127.5 (C_{ar}H), 125.6 (C_{ar}H), 123.7 (C_{ar}H), 122.6 (C_{ar}H), 120.5 (C_{ar}H), 65.8 (CH_2), 47.3 (*C*H), 44.0 (CH_2) *Z*-isomer: δ = 167.1 (*C*(O)OH), 156.8 (*C*(O)N), 154.3 (C_{ar}N), 152.3 (C_{ar}N), 144.3 (C_{ar}), 143.8 (C_{ar}) 141.2 (C_{ar}), 133.3 (C_{ar}), 132.2 (C_{ar}H), 130.4 (C_{ar}H), 128.2 (C_{ar}H), 128.0 (C_{ar}H), 127.7 (C_{ar}H), 127.5 (C_{ar}H), 125.6 (C_{ar}H), 123.1 (C_{ar}H), 122.8 (C_{ar}H), 120.5 (C_{ar}H), 65.6 (CH_2), 47.3 (*C*H), 43.5 (CH_2).

UV-Vis *E*-isomer: λ_{max} = 291 nm, 301 nm, 330 nm, 441 nm; *Z*-isomer: λ_{max} = 290 nm, 301 nm, 434 nm; isobestic point (*E*:*Z*): λ_{iso} = 386 nm.

MS (ESI+): *m/z* = 516.13 $[M+K]^+$.

Analytical data match reported literature values.[2]

2. Peptide Characterisation

2.1. Peptide sequences

Table S1: Primary sequence and solubility in 50 mM Na_2PO_4 buffer (pH 7.4) of peptides AzoPTH$_{25-37}$ and SP1-SP4.

peptide	primary sequence[a]	solubility [µM][b]
PTH$_{25-37}$	H$_2$N-^{25}RKKLQ^{30}DVHNF^{35}VAL-OH	> 1000
AzoPTH$_{25-37}$	H$_2$N-^{25}RKKLQ^{30}D-Azo-HNF^{35}VAL-OH	250
SP1	H$_2$N-^{25}RKKLQ^{30}D-Azo-VHNF^{35}VAL-OH	60
SP2	H$_2$N-^{25}RKKLQ^{30}DV-Azo-HNF^{35}VAL-OH	55
SP3	H$_2$N-^{25}RKKLQ^{30}-Azo-VHNF^{35}VAL-OH	35
SP4	H$_2$N-^{25}RKKLQ^{30}-Azo-HNF^{35}VAL-OH	25

[a] Azo = 3,4'-AMPB; [b] maximum solubility was determined *via* UV-Vis absorption spectroscopy (ε = 49310 $M^{-1}cm^{-1}$ at 205 nm for PTH$_{25-37}$, ε = 13000 $M^{-1}cm^{-1}$ at 327 nm for 3,4'-AMPB containing peptides).

2.2. HPLC- and MALDI-ToF-data

AzoPTH$_{25-37}$: MS (MALDI+): *m/z* calc. 1705.990 found 1706.254.

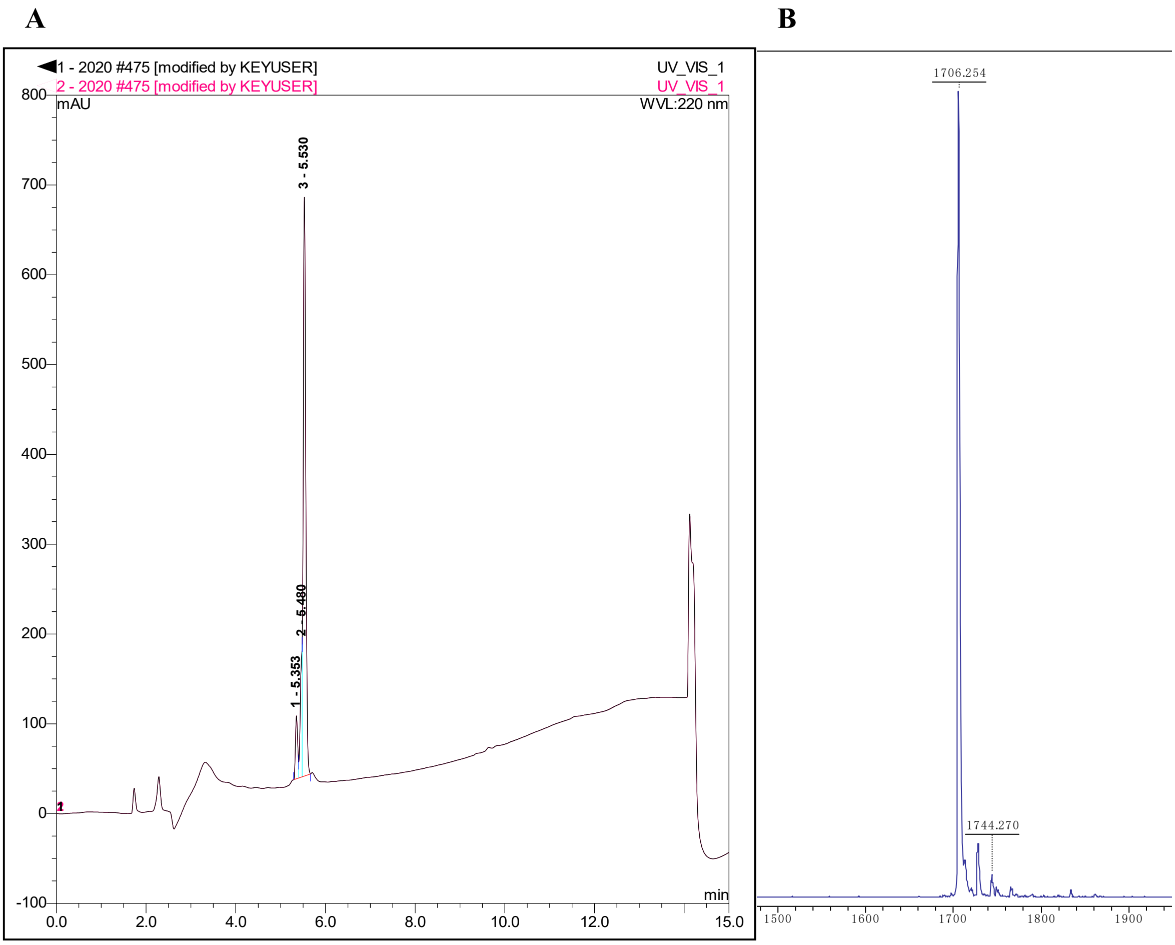

Figure S1: (**A**) HPLC-trace of AzoPTH$_{25-37}$ (*cis*-isomer at 5.353, *trans*-isomer at 5.530). (**B**) MALDI-spectrum of AzoPTH$_{25-37}$.

SP1: MS (MALDI+): *m/z* calc. 1805.123 found 1805.938.

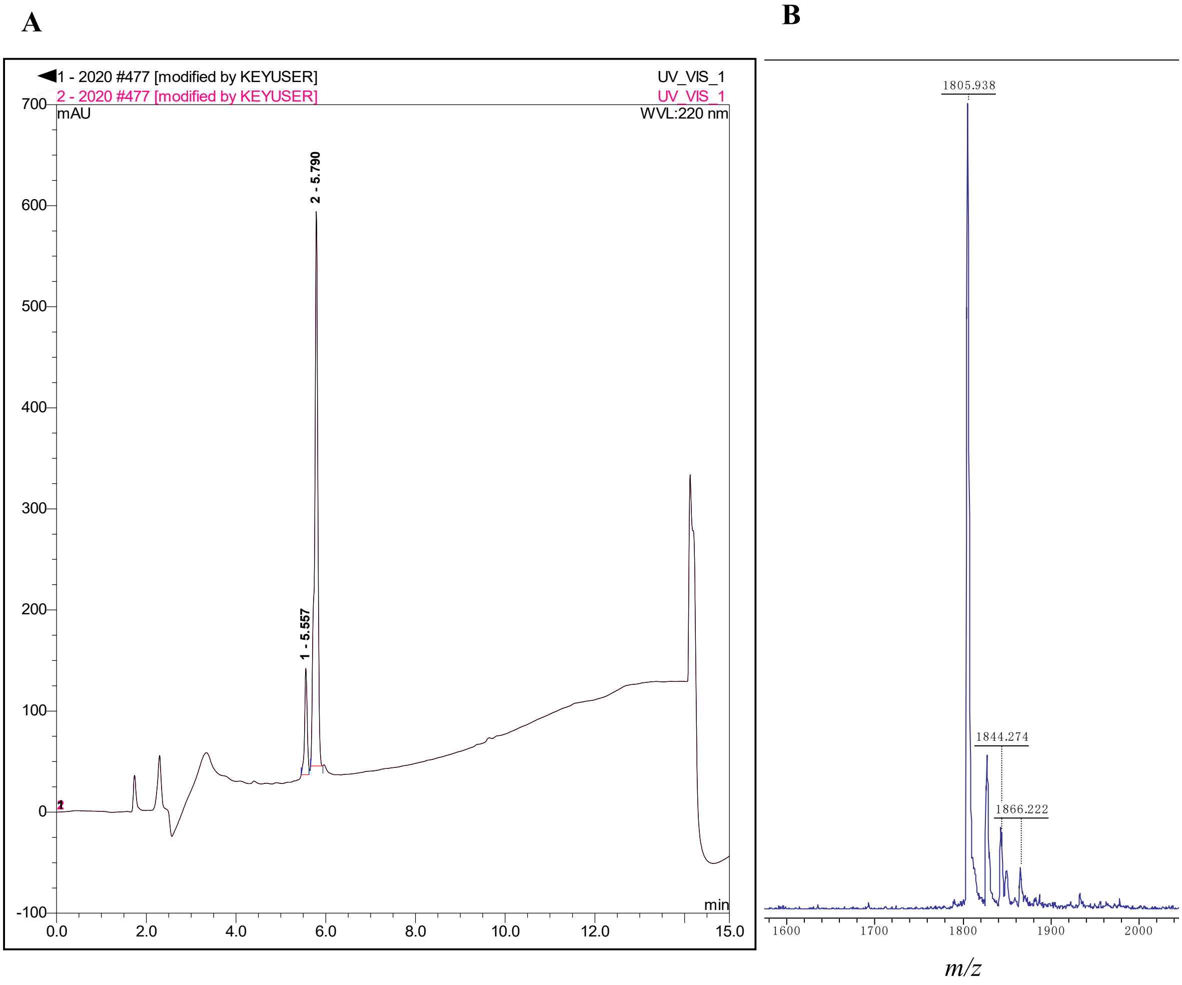

Figure S2: (**A**) HPLC-trace of SP1 (*cis*-isomer at 5.557, *trans*-isomer at 5.790). (**B**) MALDI-spectrum of SP1.

SP2: MS (MALDI+): *m/z* calc. 1805.123 found 1806.162.

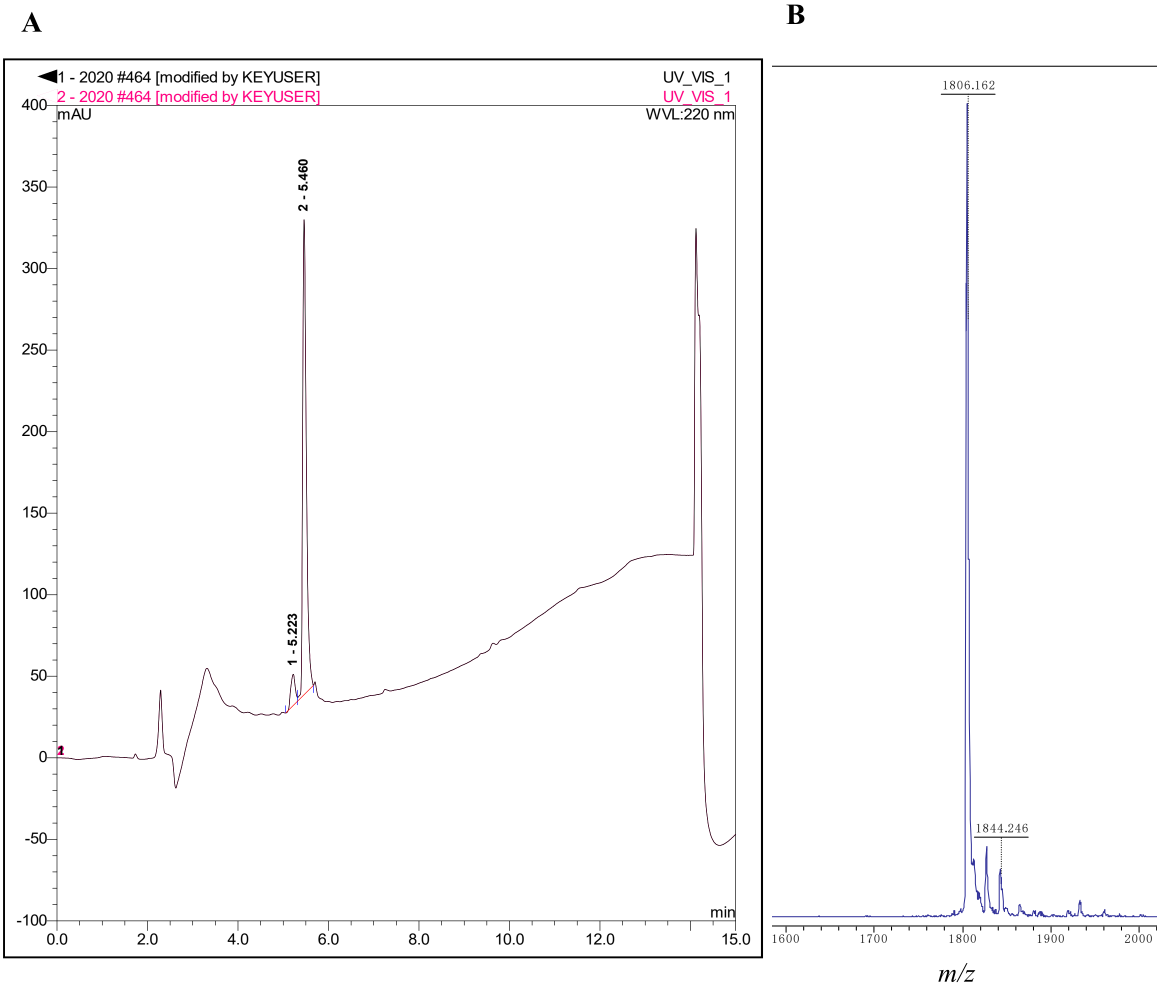

Figure S3: (**A**) HPLC-trace of SP2 (*cis*-isomer at 5.223, *trans*-isomer at 5.460). (**B**) MALDI-spectrum of SP2.

SP3: MS (MALDI+): *m/z* calc. 1690.035 found 1688.964.

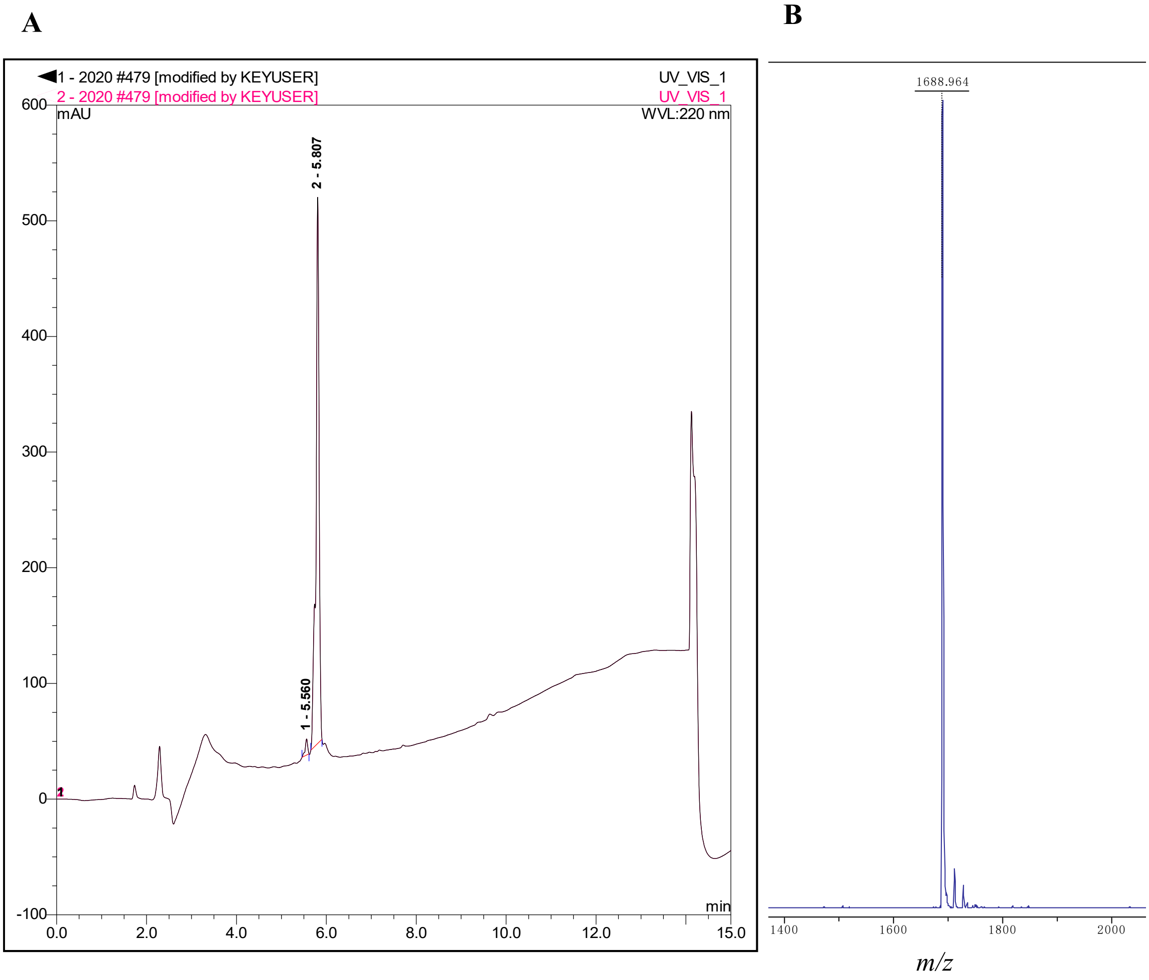

Figure S4: (**A**) HPLC-trace of SP3 (*cis*-isomer at 5.560, *trans*-isomer at 5.807). (**B**) MALDI-spectrum of SP3.

SP4: MS (MALDI+): *m/z* calc. 1590.902 found 1591.069).

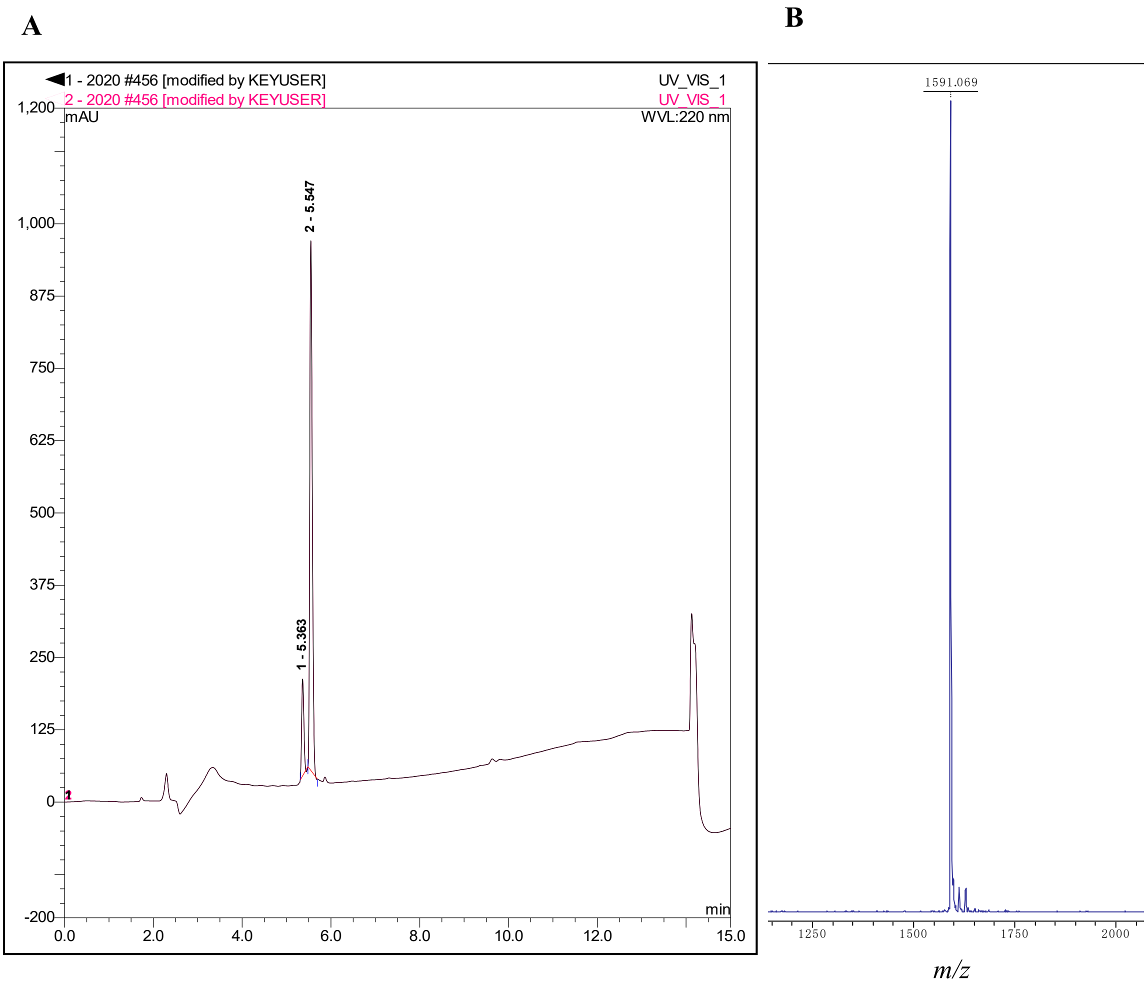

Figure S5: (**A**) HPLC-trace of SP4 (*cis*-isomer at 5.363, *trans*-isomer at 5.547). (**B**) MALDI-spectrum of SP4.

2.3. Photophysical properties of AzoPTH25-37

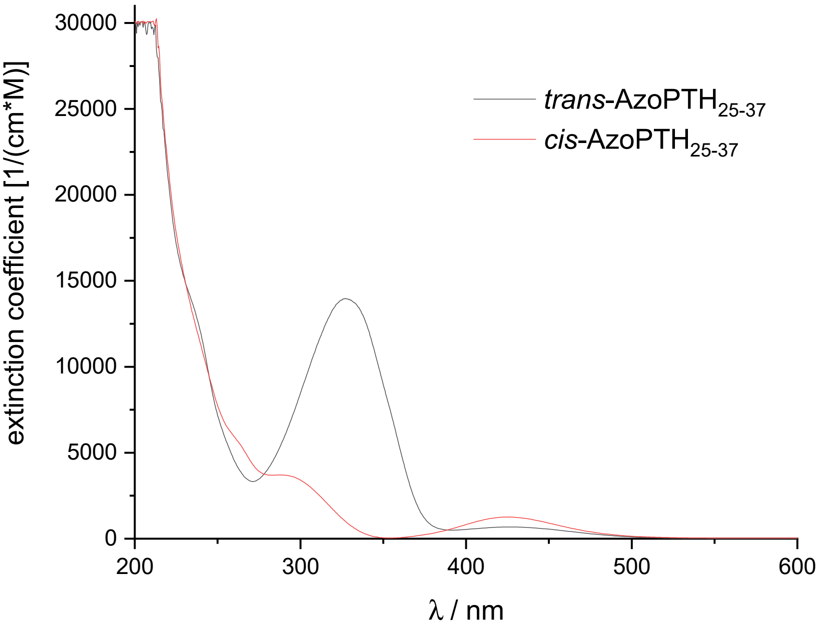

Figure S6: Separated UV/Vis-spectra of the pure isomers of AzoPTH$_{25-37}$; spectra were seperated with Wolfram Mathematica 12.2.

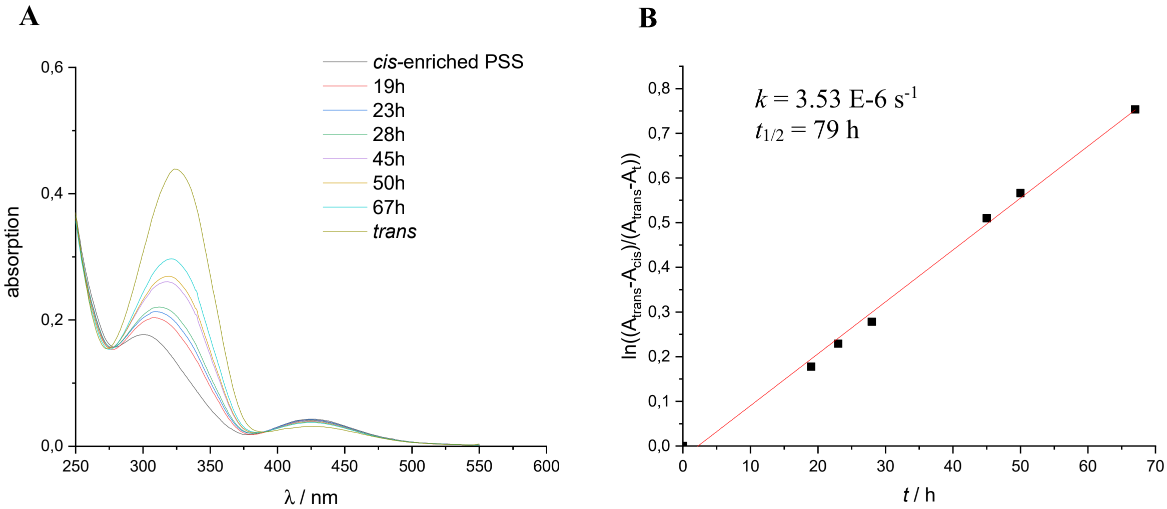

Figure S7: (**A**) UV/Vis-spectra of *trans*-isomer, *cis*-enriched PSS, and *cis*-enriched PSS sample after distinct time points in the dark. (**B**) logarithmic application of the absorption change over time to determine rate constant k and half-life time $t_{1/2}$.[2]

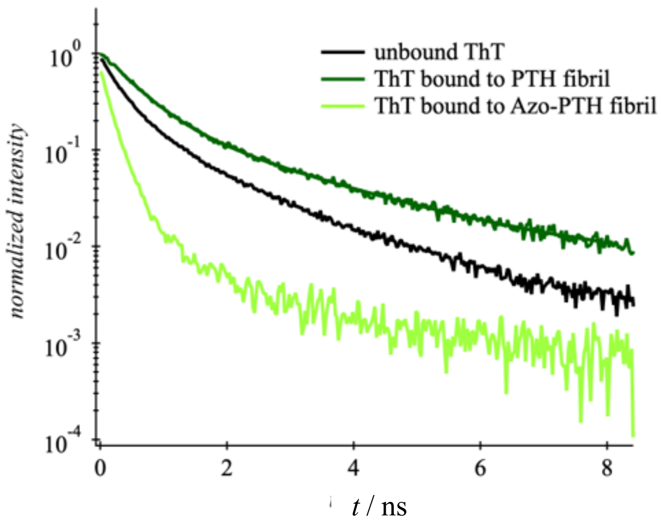

Figure S8: Time- resolved fluorescence measurement (excitation wavelength = 460 nm, emission wavelength = 480 nm) of unbound ThT (black), ThT bound to PTH_{25-37} fibrils (dark green), ThT bound to *trans*-$AzoPTH_{25-37}$ fibrils (light green).

2.4. Aggregation Kinetics of PTH_{25-37}, *trans*-$AzoPTH_{25-37}$, *cis*-$AzoPTH_{25-37}$, and mixtures

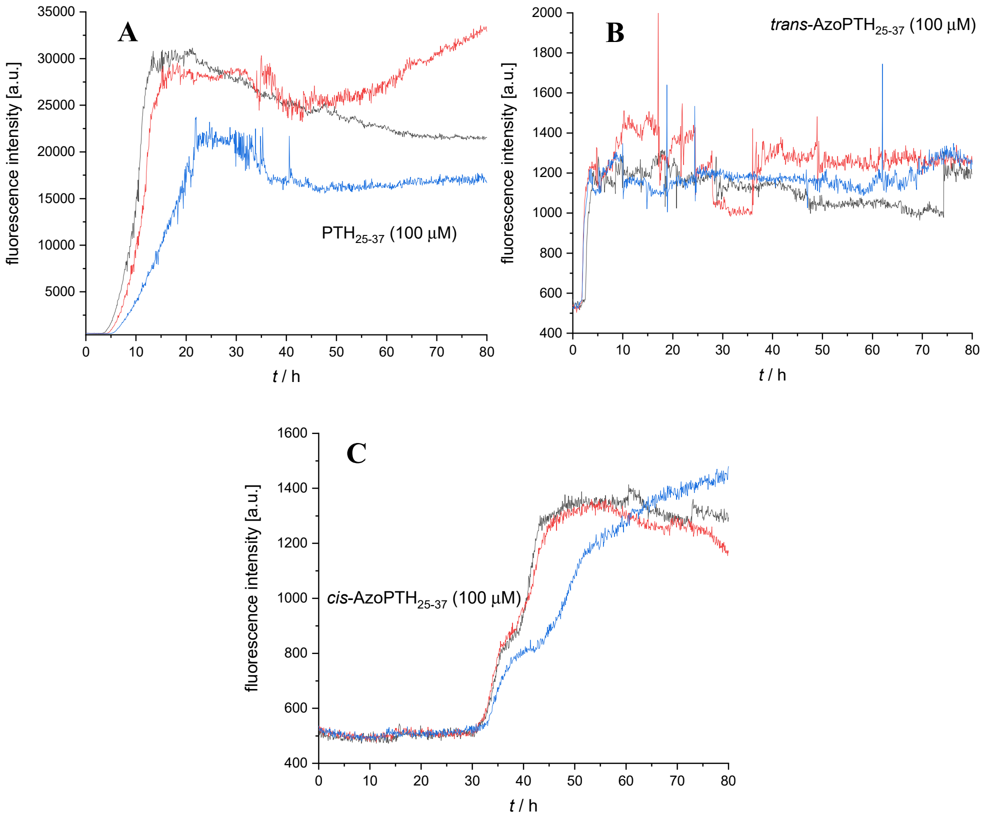

Figure S9: ThT monitored fibrillation assays (c = 100 µM, 37 °C, 50 mM Na_2HPO_4, pH7.4). (**A**) PTH_{25-37}, (**B**) *trans*-$AzoPTH_{25-37}$, (**C**) *cis*-$AzoPTH_{25-37}$.

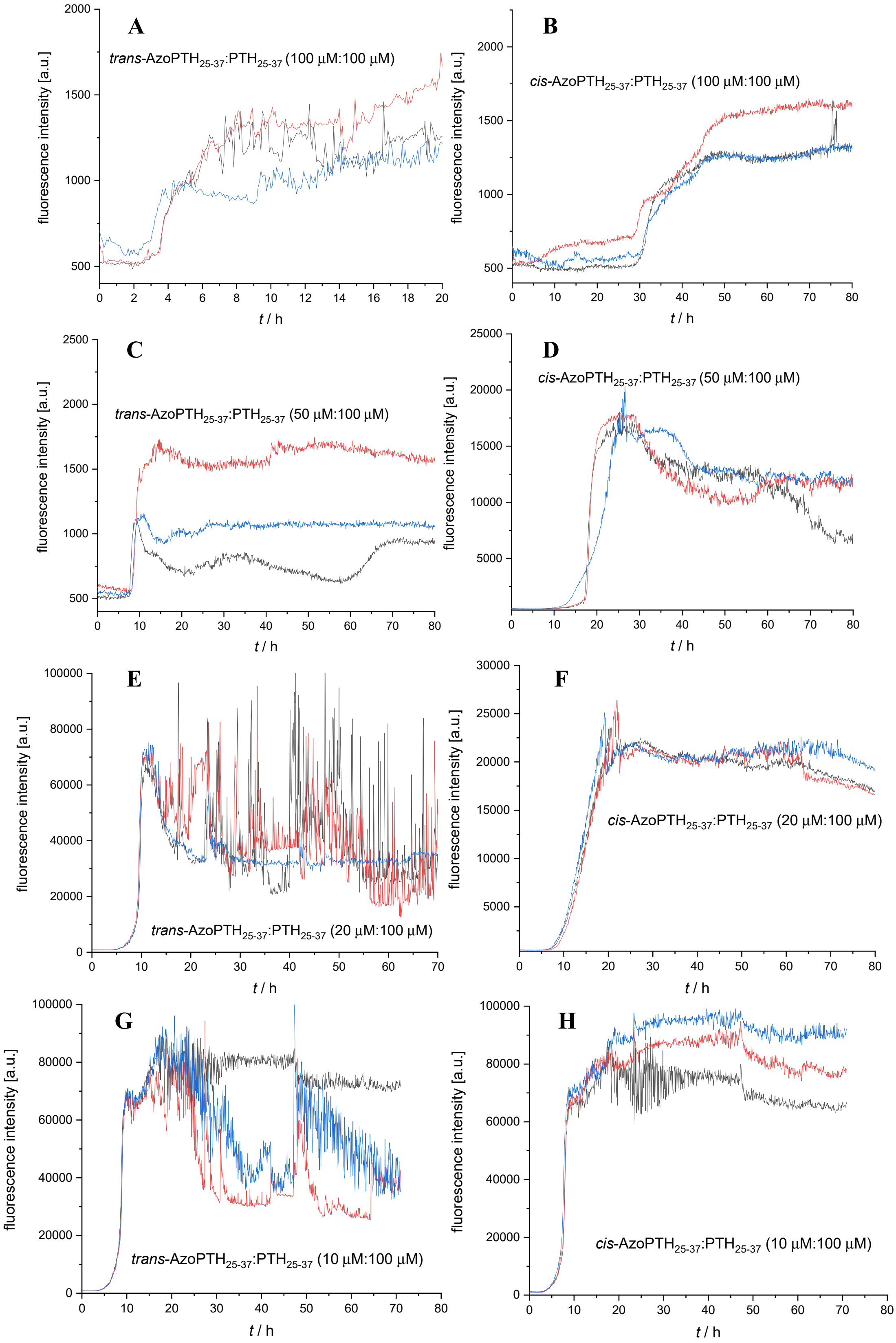

Figure S10: ThT monitored fibrillization assays of mixtures of $PTH_{25\text{-}37}$, *trans*-$AzoPTH_{25\text{-}37}$, and *cis*-$AzoPTH_{25\text{-}37}$ (37 °C, 50 mM Na_2HPO_4, pH7.4). (**A**) *trans*-$AzoPTH_{25\text{-}37}$:$PTH_{25\text{-}37}$ (100 µM:100 µM), (**B**) *cis*-$AzoPTH_{25\text{-}37}$:$PTH_{25\text{-}37}$ (100 µM:100 µM), (**C**) *trans*-$AzoPTH_{25\text{-}37}$:$PTH_{25\text{-}37}$ (50 µM:100 µM), (**D**) *cis*-$AzoPTH_{25\text{-}37}$:$PTH_{25\text{-}37}$ (50 µM:100 µM), (**E**) *trans*-$AzoPTH_{25\text{-}37}$:$PTH_{25\text{-}37}$ (20 µM:100 µM), (**F**) *cis*-$AzoPTH_{25\text{-}37}$:$PTH_{25\text{-}37}$ (20 µM:100 µM), (**G**) *trans*-$AzoPTH_{25\text{-}37}$:$PTH_{25\text{-}37}$ (10 µM:100 µM), (**H**) *cis*-$AzoPTH_{25\text{-}37}$:$PTH_{25\text{-}37}$ (10 µM:100 µM).

3. Analytical NMR-Spectra

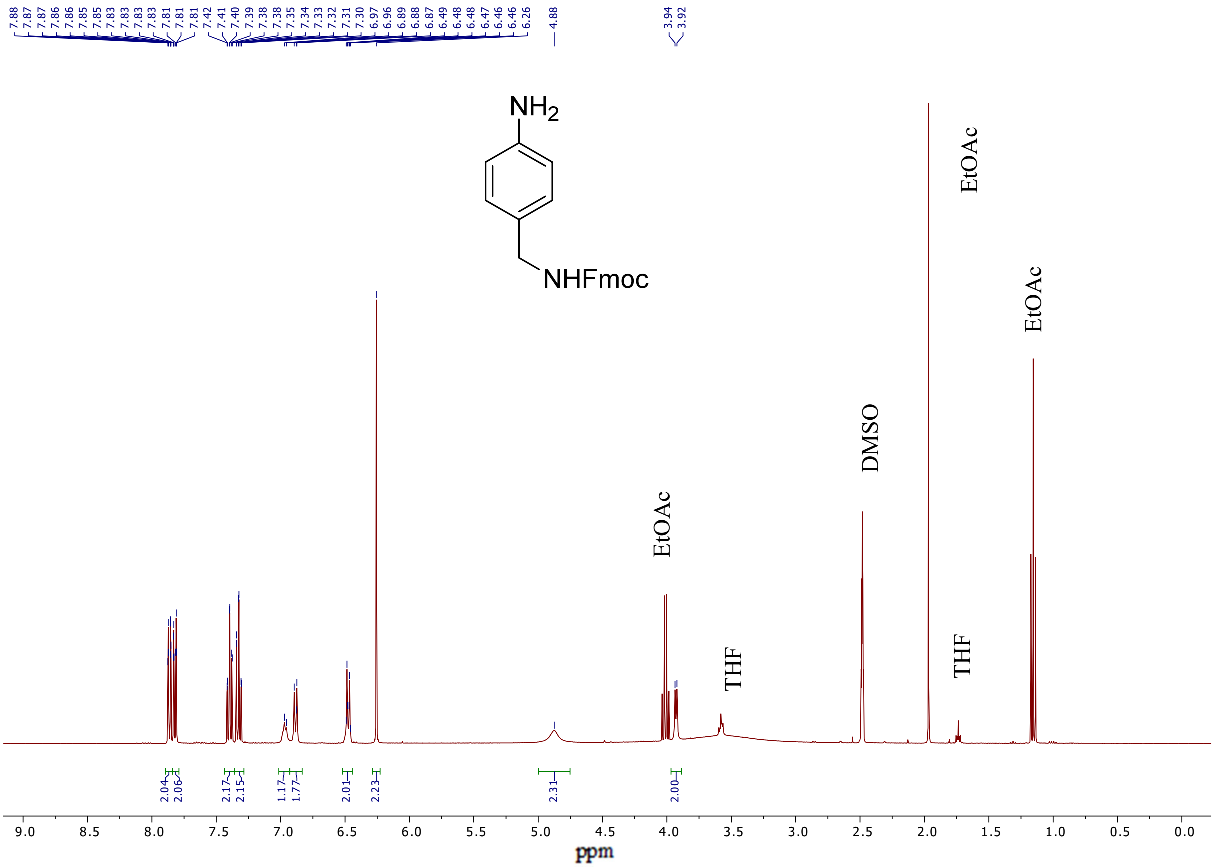

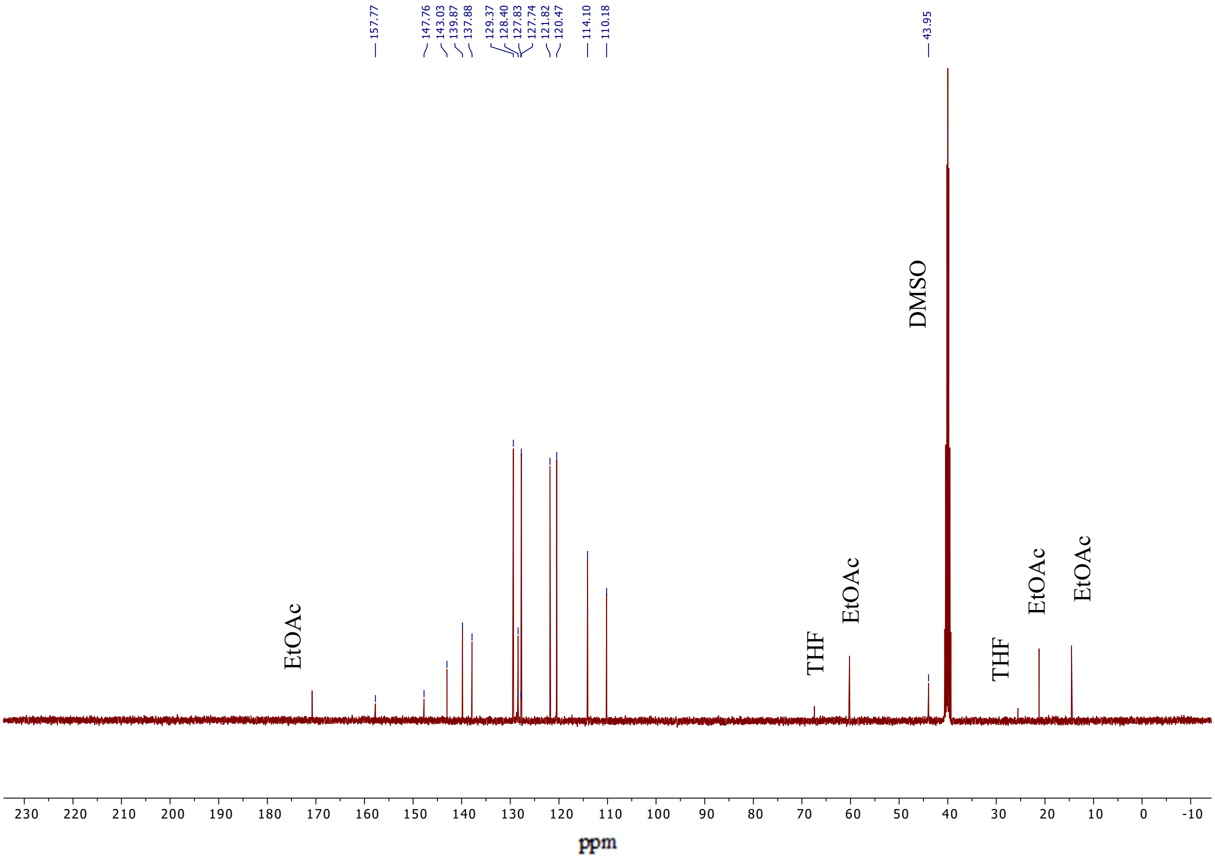

Figure S11: ^{1}H-NMR spectrum (top; 400 MHz, DMSO-d_6) and ^{13}C-NMR spectrum (bottom; 100 MHz, DMSO-d_6) of (9*H*-Fluoren-9-yl)methyl (4-aminobenzyl)carbamate.

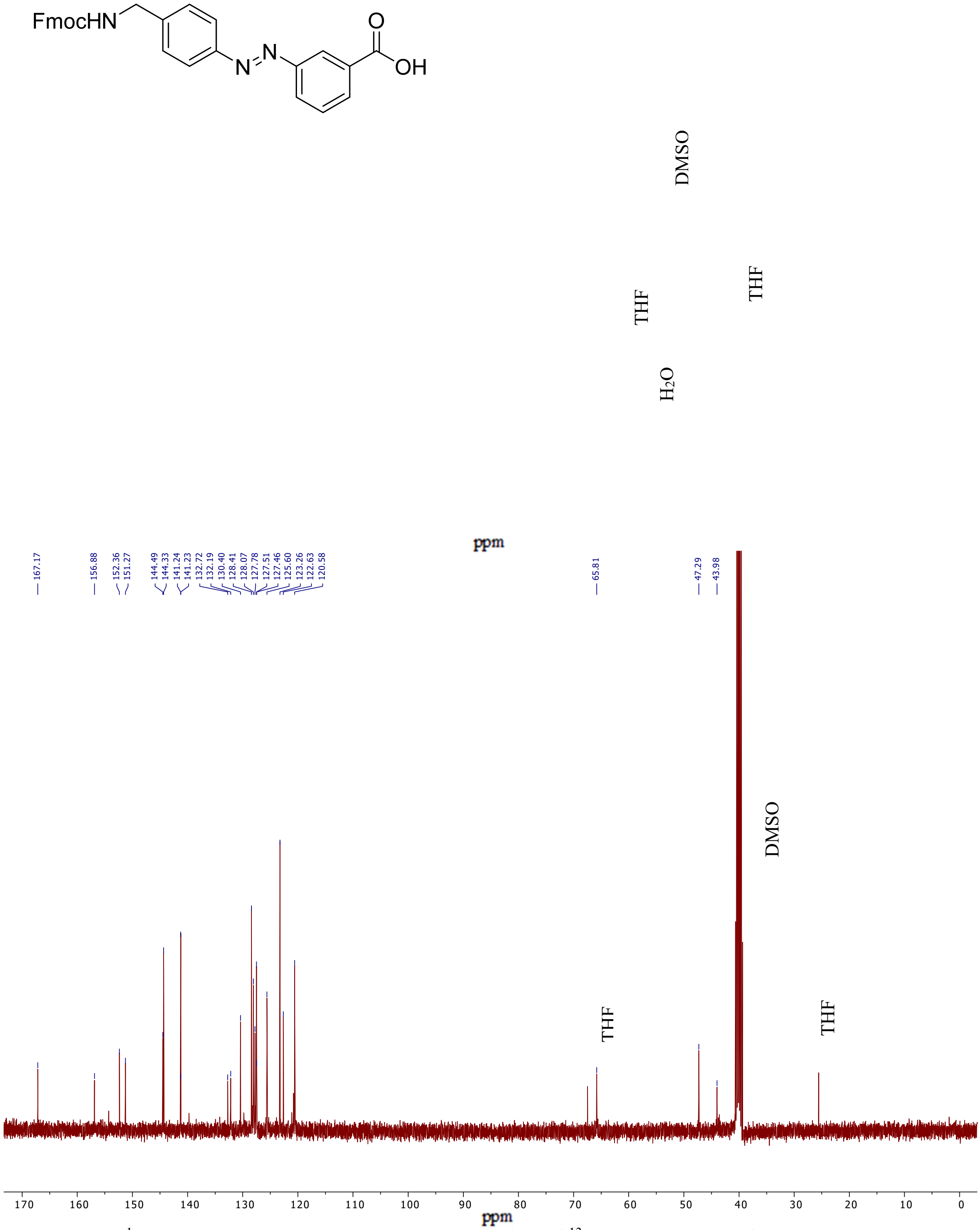

Figure S12: ^{1}H-NMR spectrum (top; 400 MHz, DMSO-d_6) and ^{13}C-NMR spectrum (bottom; 100 MHz, DMSO-d_6) of Fmoc-3,4'-AMPB (mixture of isomers).

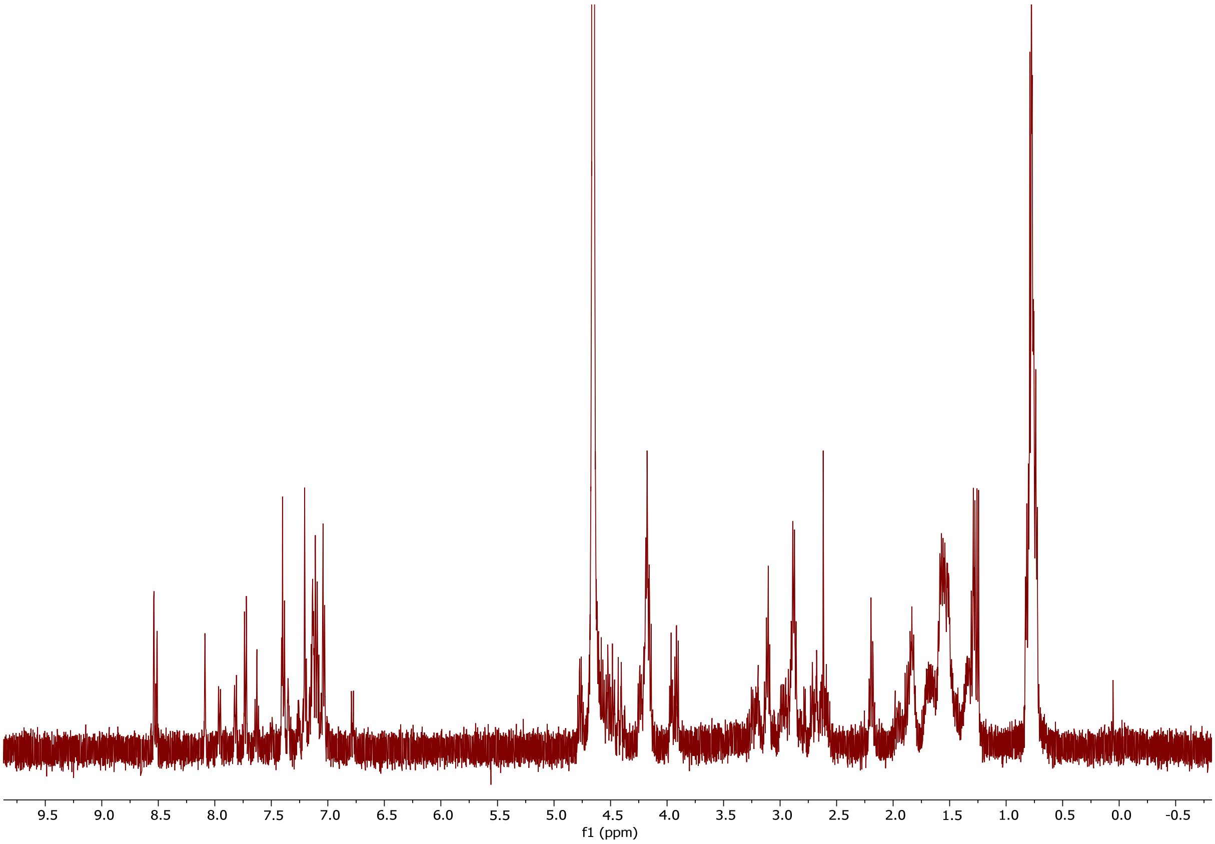

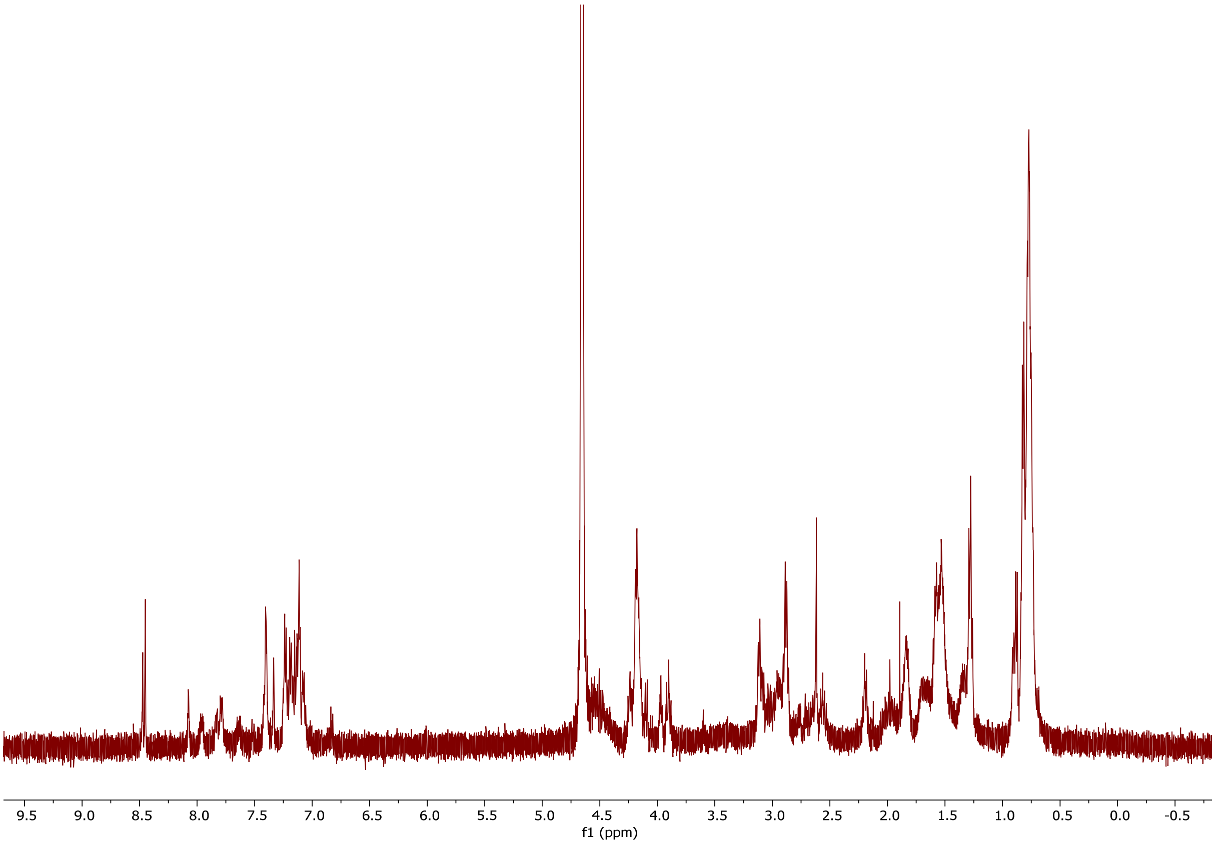

Figure S13: ^{1}H-NMR spectra (500 MHz, D_2O) of AzoPTH$_{25\text{-}37}$ (top, *trans*-isomer) and SP1 (bottom, *trans*-isomer).

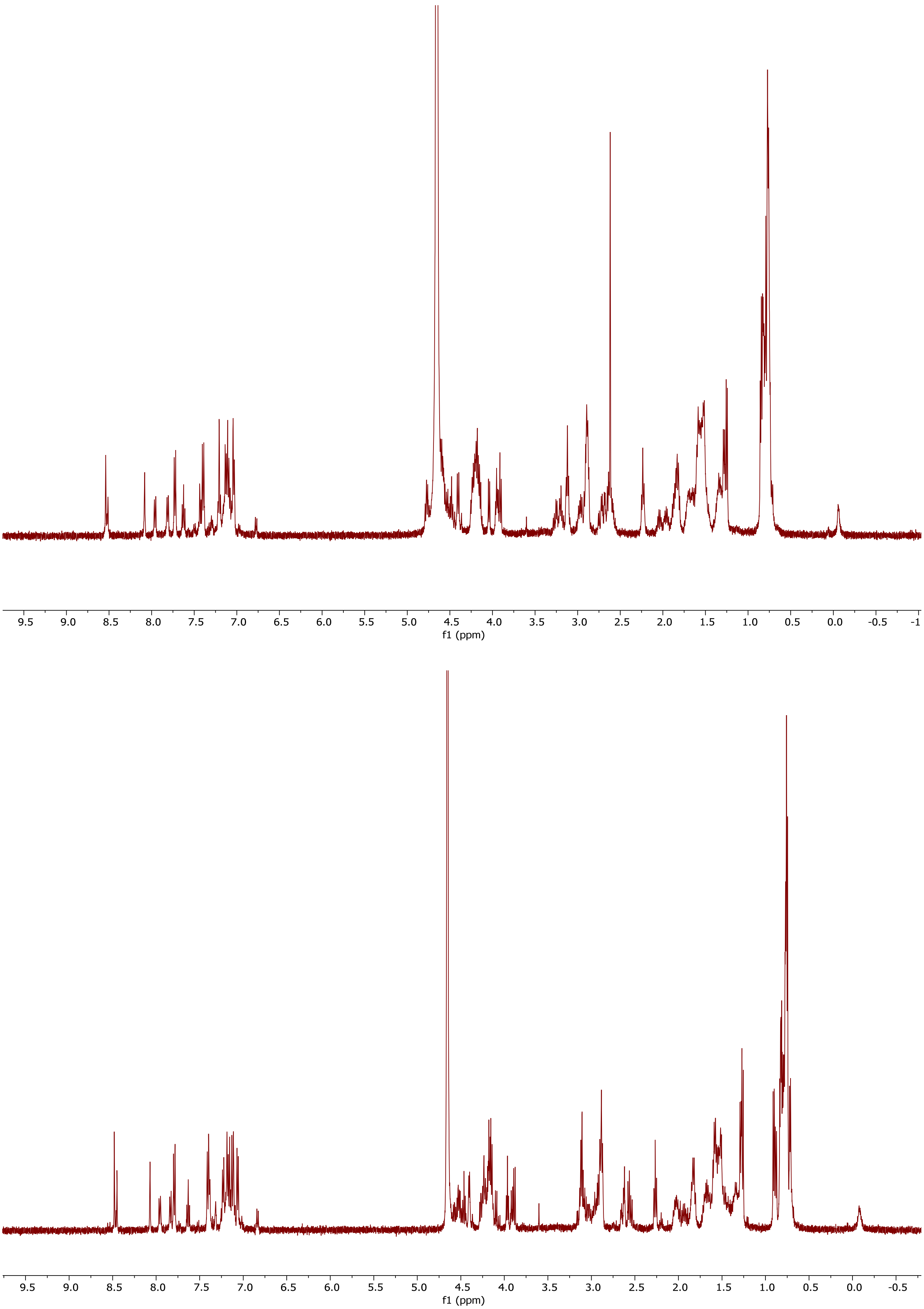

Figure S14: ^{1}H-NMR spectra (500 MHz, D_2O) of SP2 (top, *trans*-isomer) and SP3 (bottom, *trans*-isomer).

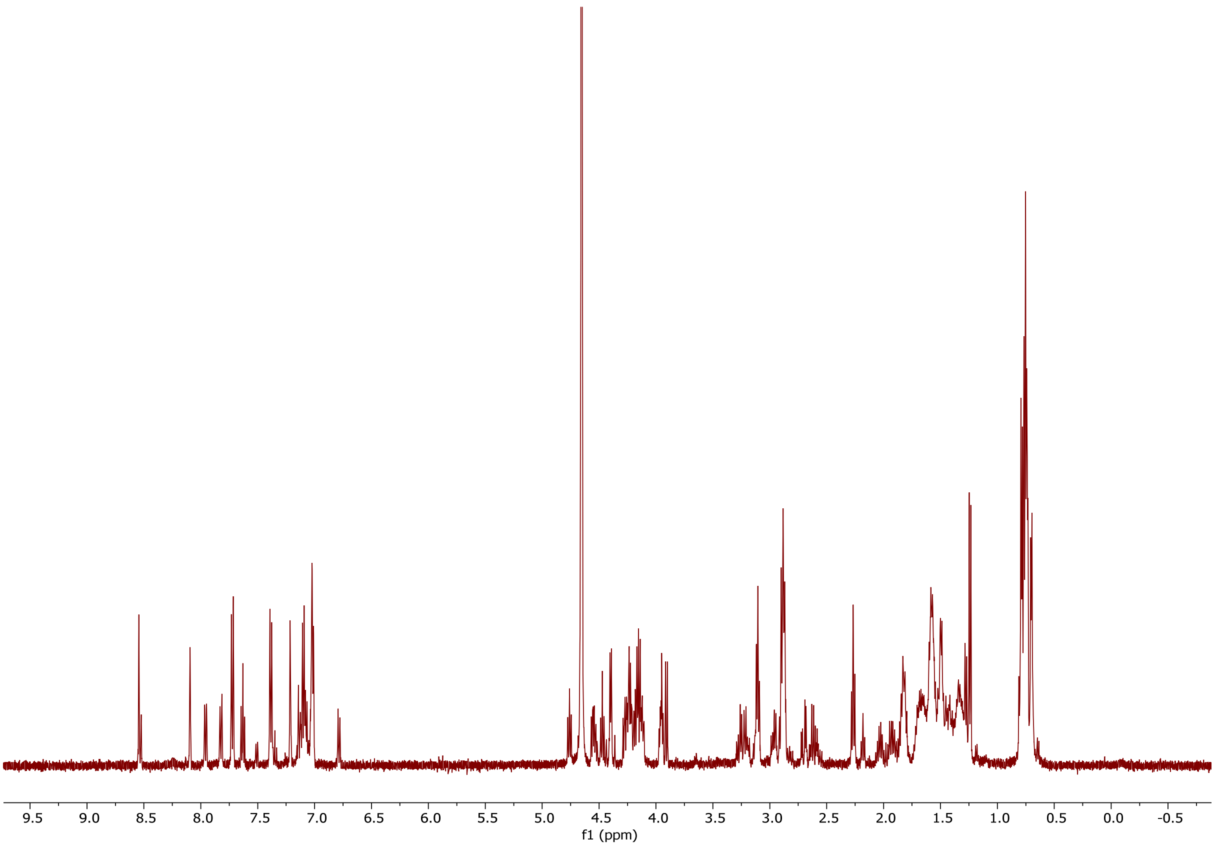

Figure S15: ^{1}H-NMR spectrum (500 MHz, D_2O) of SP4 (*trans*-isomer).

1. Murawska, G. M.; Poloni, C.; Simeth, N. A.; Szymanski, W.; Feringa, B. L., Comparative Study of Photoswitchable Zinc-Finger Domain and AT-Hook Motif for Light-Controlled Peptide–DNA Binding. *Chemistry–A European Journal* **2019,** *25* (19), 4965-4973.
2. Rück-Braun, K.; Kempa, S.; Priewisch, B.; Richter, A.; Seedorff, S.; Wallach, L., Azobenzene-Based ω-Amino Acids and Related Building Blocks: Synthesis, Properties, and Application in Peptide Chemistry. *Synthesis* **2009,** *24*, 4256-4267.
